# Needle arthroscopy as an emerging office‐based tool: Promising diagnostic value but limited evidence for therapeutic equivalence—A systematic review and frequentist meta‐analysis

**DOI:** 10.1002/jeo2.70857

**Published:** 2026-07-30

**Authors:** Ingo J. Banke, Roland Becker, Maximilian Voss, Nikolai Ramadanov

**Affiliations:** ^1^ Clinic of Orthopaedics and Sports Orthopaedics, School of Medicine and Health TUM University Hospital, Technical University of Munich Munich Germany; ^2^ AGA‐Society for Arthroscopy and Joint‐Surgery Hip Committee, c/o Walder Wyss Ltd. Zurich Switzerland; ^3^ Center of Orthopaedics and Traumatology, Brandenburg Medical School University Hospital Brandenburg Brandenburg an der Havel Germany; ^4^ Faculty of Health Science Brandenburg Brandenburg Medical School Theodor Fontane Brandenburg an der Havel Germany

**Keywords:** arthroscopy, diagnostic accuracy, In‐office arthroscopy, Nano‐arthroscopy, needle arthroscopy

## Abstract

**Purpose:**

Needle (Nano) arthroscopy is an emerging minimally invasive technique primarily used for diagnostic assessment and selected therapeutic procedures that may facilitate office‐based joint evaluation. However, comparative evidence regarding functional outcomes, procedural performance and safety remains limited. This systematic review and meta‐analysis compared needle arthroscopy with conventional arthroscopy regarding functional outcomes, diagnostic performance and complication rates.

**Methods:**

Comparative studies of needle arthroscopy versus conventional arthroscopy were pooled using random‐effects meta‐analysis with Hartung–Knapp adjustment. Dichotomous outcomes were analysed as odds ratios (ORs) and continuous outcomes as mean differences (MDs), both with 95% confidence intervals (CIs). Functional outcomes, procedural success, complications and diagnostic performance were quantitatively synthesized. Between‐study heterogeneity was assessed using *I* ^2^ and *τ* ^2^, and diagnostic parameters were analysed exploratorily when required data were reconstructed from reported percentages.

**Results:**

Nine studies including 503 patients were analysed. Needle arthroscopy and conventional arthroscopy demonstrated comparable procedure completion (OR 1.00, 95% CI 0.17–5.93) and diagnostic success (OR 1.01, 95% CI 0.10–10.07). Complication rates were low and similar between groups (OR 1.00, 95% CI 0.20–5.06), with no differences in infection or reoperation. Needle arthroscopy showed improved functional outcomes at 0.5 months (KOOS4 [KOOS composite score (mean of four subscales)]: MD +15.25, 95% CI 7.82–22.67) and maintained a significant advantage at 1.5 months (MD +7.25, 95% CI 0.27–14.22). Exploratory diagnostic analyses revealed no significant differences in sensitivity or specificity. Certainty of evidence ranged from moderate to very low.

**Conclusion:**

Needle arthroscopy provides comparable diagnostic and procedural performance to conventional arthroscopy and offers an early functional advantage. However, while current evidence is insufficient to replace conventional arthroscopy as the reference standard, needle arthroscopy may represent a valuable adjunct or emerging alternative in selected clinical settings.

**Level of Evidence:**

Level III, systematic review and meta‐analysis of predominantly retrospective cohort studies.

## Graphical abstract

Needle (Nano) arthroscopy provides substantially smaller access than conventional arthroscopy and may facilitate office‐based procedures. In this systematic review and meta‐analysis of nine studies comprising 503 patients, pooled estimates showed no significant differences in procedure completion, diagnostic success, or complication rates, while needle arthroscopy was associated with improved early functional recovery. It represents a promising diagnostic adjunct; however, therapeutic equivalence remains unproven, and conventional arthroscopy remains the reference standard. KOOS4, Knee injury and Osteoarthritis Outcome Score composite; OR, odds ratio.

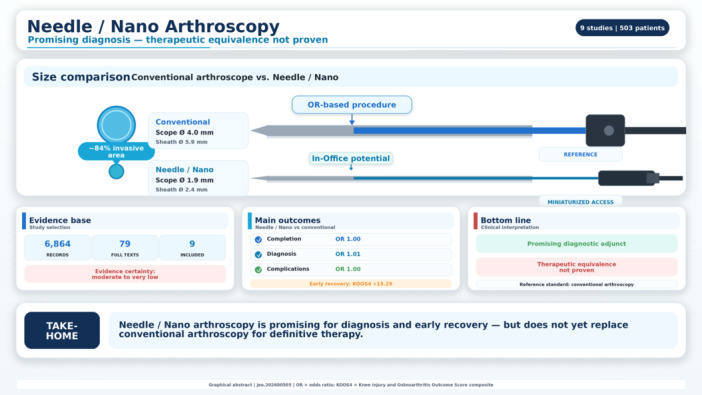

**Abbreviations**
AS: arthroscopy (conventional arthroscopy)
CI: confidence interval
FN: false negative
FP: false positive
GRADE: Grading of Recommendations Assessment, Development and Evaluation
*I* ^2^
: heterogeneity statistic
IONA: In‐office needle arthroscopy
KOOS: Knee injury and Osteoarthritis Outcome Score
KOOS4: KOOS composite score (mean of four subscales)
LoE: level of evidence
MD: mean difference
MRI: magnetic resonance imaging
Nano: Needle (Nano) arthroscopy
OR: odds ratio
PRISMA: Preferred Reporting Items for Systematic Reviews and Meta‐Analyses
PROSPERO: International Prospective Register of Systematic Reviews
RCT: randomized controlled trial
REML: restricted maximum likelihood
ROBINS‐I: Risk Of Bias In Non‐randomized Studies of Interventions
RoB 2: Risk of Bias 2 tool (for randomized trials)
SD: standard deviation
TN: true negative
TP: true positive
*τ* ^2^
: between‐study variance

## INTRODUCTION

Arthroscopy has fundamentally transformed the diagnosis and treatment of intra‐articular pathology by enabling minimally invasive visualization and intervention with high diagnostic and therapeutic precision. Ongoing technological advances have further reduced procedural invasiveness while expanding clinical applicability across multiple joints.

Needle arthroscopy, also referred to as Nano arthroscopy, represents a further evolution of minimally invasive joint surgery. By utilizing ultra‐small‐diameter arthroscopes introduced through needle‐sized portals, these systems allow direct intra‐articular visualization with minimal soft‐tissue disruption. Contemporary commercially available platforms typically range from 1.9 to 2.3 mm in diameter and include systems such as the Arthrex NanoScope™, Trice Medical mi‐eye 3 and IntraVu 30 SideVu®. The introduction of in‐office needle arthroscopy (IONA) has further expanded potential applications by enabling selected procedures under local anaesthesia in outpatient settings [10, 96].

Recent literature has demonstrated promising diagnostic performance of Needle arthroscopy across multiple joints, with several studies reporting high sensitivity and specificity for intra‐articular pathology and, in selected indications, diagnostic accuracy comparable or even superior to magnetic resonance imaging (MRI) [10, 11, 51, 96]. In addition to diagnostic utility, Needle arthroscopy has been associated with potential practical advantages, including rapid in‐office assessment, avoidance of general anaesthesia, reduced operating‐room utilization, shorter diagnostic pathways and favourable patient satisfaction [51]. Preliminary therapeutic applications have also been described for meniscal surgery, ankle impingement, shoulder procedures, fracture‐assisted reduction techniques and second‐look biological assessment following anterior cruciate ligament repair and reconstruction [53, 56, 76, 93]. Furthermore, safety data appear encouraging, with a recent systematic review reporting exclusively minor complications following Needle arthroscopy procedures [89].

However, despite increasing clinical adoption and expanding indications, the currently available evidence remains limited and heterogeneous. Most published studies consist of small case series, feasibility investigations or observational cohorts with variable methodology and inconsistent outcome reporting. Existing systematic reviews are predominantly descriptive and lack robust quantitative comparisons with conventional arthroscopy [10, 51, 89, 96]. More broadly, recent umbrella evidence has demonstrated that, despite the increasing number of evidence syntheses in sports orthopaedics, the methodological quality of meta‐analyses remains variable, underscoring the need for rigorous quantitative evidence synthesis [69]. Consequently, the overall clinical effectiveness, diagnostic reliability, safety profile and potential therapeutic role of Needle arthroscopy remain incompletely defined.

Therefore, a rigorous comparative quantitative synthesis is warranted. The aim of this systematic review and frequentist meta‐analysis was to compare Needle arthroscopy with conventional arthroscopy regarding functional outcomes, diagnostic and procedural performance and complication rates. Furthermore, diagnostic accuracy parameters and their potential impact on clinical decision‐making were explored to better define the current role of Needle arthroscopy in clinical practice. We hypothesized that Needle arthroscopy would demonstrate comparable diagnostic and procedural performance to conventional arthroscopy while providing improved short‐term functional recovery and similar complication rates.

## METHODS

### Study design and registration

This study was registered in International Prospective Register of Systematic Reviews (PROSPERO) on 9 March 2026 (registration number: CRD420261335973). The review was conducted in accordance with the Preferred Reporting Items for Systematic Reviews and Meta‐Analyses (PRISMA) 2020 guidelines [64]. The completed PRISMA 2020 checklist is provided in Table S1.

### Search strategy

A comprehensive literature search was performed in five electronic databases: PubMed/MEDLINE, Embase, Scopus, Epistemonikos and the Cochrane Central Register of Controlled Trials (CENTRAL). The final search date was 31 March 2026.

The search strategy combined free‐text terms related to Needle arthroscopy and Nano‐arthroscopy, including but not limited to: ‘needle arthroscopy’, ‘nano arthroscopy’, ‘nanoarthroscopy’, ‘nanoscopic arthroscopy’, ‘needle arthroscope’, ‘in‐office arthroscopy’, ‘office‐based arthroscopy’, ‘outpatient needle arthroscopy’, ‘NanoScope’, ‘NanoNeedle Scope’, ‘Arthrex NanoScope’ and ‘Arthrex NanoNeedleScope’. Database‐specific syntax was adapted for each platform.

Reference lists of included studies and relevant reviews were manually screened (backward citation tracking), and forward citation searches were performed using Scopus and Google Scholar. No restrictions on study design were applied during the search phase.

### Eligibility criteria

Studies were included if they met the following criteria: (1) Primary clinical studies evaluating Needle arthroscopy, Nano arthroscopy or IONA; (2) reporting clinical outcomes, diagnostic performance, procedural characteristics or complications and (3) prospective or retrospective cohort studies, comparative studies or randomized controlled trials (RCTs). The following were excluded: (1) Editorials, narrative reviews, systematic reviews and meta‐analyses; (2) animal or cadaveric studies; (3) single case reports; (4) conference abstracts without full text; (5) non‐English publications and (6) study screening and selection. All records were imported into reference management software and duplicates were removed. Two independent reviewers screened studies in two stages (title/abstract and full‐text). Disagreements were resolved by consensus. Inter‐reviewer agreement was quantified using Cohen's *κ*.

### Data extraction

Two reviewers independently extracted data using a predefined and piloted extraction sheet. Extracted variables included: (1) Study characteristics (author, year, country, study design); (2) patient characteristics (sample size, age, sex distribution); (3) joint and clinical indication; (4) type of procedure (diagnostic vs. therapeutic); (5) procedural setting (in‐office vs. operating room); (6) anaesthesia type; (7) reported outcomes; (8) complications and (9) follow‐up duration. Where available, patient‐reported outcome measures (PROMs) were extracted. Given the heterogeneity of PROM reporting across studies, analysis focused on Knee injury and Osteoarthritis Outcome Score (KOOS) composite score (mean of four subscales) (KOOS4) or KOOS‐derived composite scores when available.

### Outcome measures

The primary outcomes of interest were: (I) Procedural and clinical outcomes: (1) procedure completion rate; (2) successful diagnosis; (3) any complication; (4) minor complication; (5) infection; (6) reoperation and (7) management change. (II) Diagnostic performance outcomes: (1) true positive (TP); (2) true negative (TN); (3) false positive (FP); (4) false negative (FN); (5) sensitivity and (6) specificity. (III) Patient‐reported outcomes: (1) KOOS4 (baseline) and (2) KOOS4 (short‐term follow‐up, where available).

### Risk of bias assessment

Risk of bias was assessed at the study level using the Risk Of Bias In Non‐randomized Studies of Interventions (ROBINS‐I) tool [77] for non‐randomized studies and the Risk of Bias 2 tool (RoB 2) [78] for the RCT. For non‐randomized studies, the ROBINS‐I [77] domains included bias due to confounding, participant selection, classification of interventions, deviations from intended interventions, missing data, outcome measurement and selection of the reported result. Each domain was judged as low, moderate, serious or critical risk of bias, and an overall study‐level judgement was assigned accordingly. For the randomized trial, the RoB 2 [78] was applied, evaluating bias arising from the randomization process, deviations from intended interventions, missing outcome data, measurement of the outcome and selection of the reported result. Each domain was rated as low risk, some concerns or high risk of bias. All assessments were performed at the study level, and disagreements were resolved by consensus.

### Certainty of evidence assessment

The certainty of evidence was assessed using the Grading of Recommendations Assessment, Development and Evaluation (GRADE) [30] approach for each outcome separately across the domains of risk of bias, inconsistency, indirectness, imprecision and publication bias. Non‐randomized studies were initially rated as low certainty and downgraded where appropriate, while the randomized trial contributed to upgrading where justified.

Imprecision was rated as serious in the presence of wide confidence intervals (CIs) or few events. Diagnostic accuracy outcomes (TP, TN, FP, FN, sensitivity, specificity) were reconstructed from reported percentages and were therefore considered indirect and imprecise.

### Statistical analysis

A pairwise meta‐analysis was performed to compare Needle arthroscopy with conventional arthroscopy (AS) across all outcomes reported in at least two studies. Continuous outcomes were pooled as mean differences (MDs) with corresponding 95% CIs, whereas dichotomous outcomes were analysed using odds ratios (ORs) with 95% CI.

For continuous data (KOOS4), inverse‐variance weighting was applied. For dichotomous outcomes (e.g., complications, successful diagnosis, procedure completion, management change), log‐transformed ORs were calculated using a continuity correction of 0.5 in studies with zero‐event cells.

All analyses were conducted using a random‐effects model based on restricted maximum likelihood (REML), with Hartung–Knapp adjustment [70] applied to account for between‐study uncertainty. Statistical heterogeneity was assessed using the *I* ^2^ statistic, Cochran's *Q* test and *τ* ^2^ [85].

Given the inconsistent reporting of diagnostic accuracy data across studies, including heterogeneous reference standards and incomplete 2 × 2 contingency tables, diagnostic parameters (TP, TN, FP, FN, sensitivity and specificity) were reconstructed from reported percentages and total sample sizes where necessary. These analyses were therefore considered exploratory.

Forest plots were generated for all outcomes, displaying study‐level effect estimates, corresponding CIs and study weights, along with pooled estimates. Funnel plots were constructed for outcomes with sufficient studies (≥3) to assess potential publication bias [23]. All analyses were performed using custom statistical scripts in Python.

## RESULTS

### Search results

**Figure 1:**
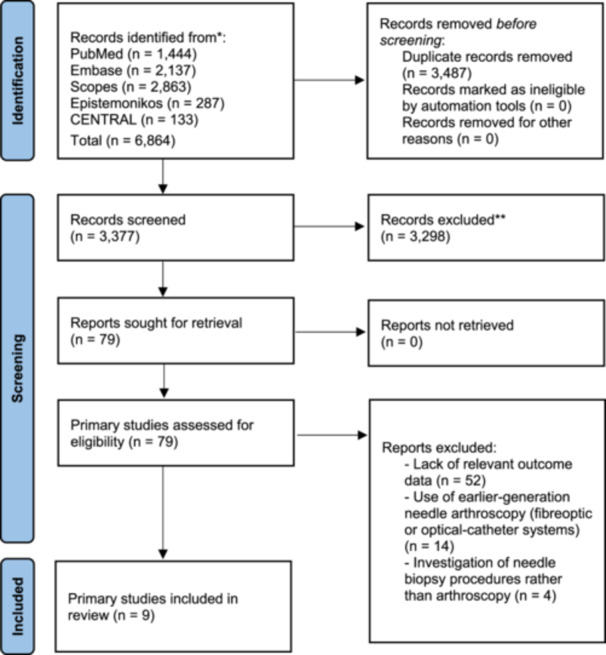
PRISMA flow diagram illustrating the study selection process. A total of 6864 records were identified through database searching. After removal of 3487 duplicates, 3377 records were screened, of which 3298 were excluded. Seventy‐nine full‐text articles [1, 2, 3, 4, 5, 6, 7, 8, 9, 12, 13, 14, 15, 16, 17, 18, 19, 20, 21, 22, 24, 25, 26, 27, 28, 29, 31, 32, 33, 34, 35, 36, 37, 38, 39, 40, 41, 42, 43, 44, 45, 46, 47, 48, 49, 50, 52, 54, 55, 57, 58, 59, 60, 61, 62, 63, 65, 66, 67, 68, 71, 72, 73, 74, 75, 79, 80, 81, 82, 83, 84, 86, 87, 88, 90, 91, 92, 94, 95] were assessed for eligibility, and 70 were excluded [2, 3, 4, 5, 6, 7, 8, 12, 13, 15, 16, 17, 18, 20, 21, 22, 24, 25, 26, 27, 28, 29, 31, 32, 33, 34, 35, 36, 37, 38, 39, 40, 41, 42, 43, 44, 46, 47, 48, 49, 50, 52, 54, 57, 58, 59, 60, 62, 63, 65, 66, 67, 68, 71, 72, 73, 74, 79, 80, 81, 82, 83, 84, 86, 87, 90, 91, 92, 94, 95]. Ultimately, nine studies [1, 9, 14, 19, 45, 55, 61, 75, 88] were included in the qualitative and quantitative synthesis. PRISMA, Preferred Reporting Items for Systematic Reviews and Meta‐Analyses.

The database search identified a total of 6864 records, including 1444 from PubMed/MEDLINE, 2137 from Embase, 2863 from Scopus, 287 from Epistemonikos and 133 from the CENTRAL. After removal of 3487 duplicates, 3377 records remained for title and abstract screening. Of these, 3298 were excluded based on predefined eligibility criteria. A total of 79 full‐text articles [1, 2, 3, 4, 5, 6, 7, 8, 9, 12, 13, 14, 15, 16, 17, 18, 19, 20, 21, 22, 24, 25, 26, 27, 28, 29, 31, 32, 33, 34, 35, 36, 37, 38, 39, 40, 41, 42, 43, 44, 45, 46, 47, 48, 49, 50, 52, 54, 55, 57, 58, 59, 60, 61, 62, 63, 65, 66, 67, 68, 71, 72, 73, 74, 75, 79, 80, 81, 82, 83, 84, 86, 87, 88, 90, 91, 92, 94, 95] were assessed for eligibility, of which 70 were excluded [2, 3, 4, 5, 6, 7, 8, 12, 13, 15, 16, 17, 18, 20, 21, 22, 24, 25, 26, 27, 28, 29, 31, 32, 33, 34, 35, 36, 37, 38, 39, 40, 41, 42, 43, 44, 46, 47, 48, 49, 50, 52, 54, 57, 58, 59, 60, 62, 63, 65, 66, 67, 68, 71, 72, 73, 74, 79, 80, 81, 82, 83, 84, 86, 87, 90, 91, 92, 94, 95]. Among the excluded studies, 52 [2, 3, 4, 5, 6, 8, 12, 13, 15, 16, 17, 18, 21, 22, 24, 25, 27, 28, 33, 36, 37, 38, 39, 40, 41, 42, 43, 44, 48, 49, 50, 57, 58, 59, 60, 63, 65, 66, 67, 68, 71, 72, 73, 74, 79, 80, 81, 82, 90, 91, 92, 95] did not report relevant outcomes, 14 [7, 20, 29, 31, 32, 34, 35, 52, 54, 62, 83, 86, 87, 94] evaluated earlier generations of needle arthroscopy (fibreoptic or optical‐catheter systems) and 4 [26, 46, 47, 84] investigated needle biopsy procedures rather than arthroscopy. Ultimately, nine needle arthroscopy studies [1, 9, 14, 19, 45, 55, 61, 75, 88] were included in the final qualitative and quantitative synthesis. The study selection process is illustrated in Figure 1.

### Study characteristics and procedural details

Across the nine included studies, a total of 503 patients were analysed, with sample sizes ranging from 7 to 198 patients per study. Seven studies investigated knee pathology, whereas two studies focused on shoulder indications. Study designs were predominantly non‐randomized comparative cohorts, with one RCT and two feasibility or economic evaluations. Levels of evidence ranged from I to V. The mean age of participants ranged from approximately 42–56 years, with a relatively balanced sex distribution across studies. Reported follow‐up periods ranged from approximately 1–3 months (Table 1).

**Table 1:** Study and patient characteristics of the included studies.

First author	Year	Journal	Country	Study period	Study design	Procedure	LoE	Total patients, *N*	Total joints, *N*	Mean age ± SD (range)	Female %	BMI mean ± SD (range)	Mean follow‐up (range)
Abbas	2023	*Journal of Orthopaedic Surgery and Research*	Canada	2016–2020	Economic modelling study/financial analysis	Nano	V	198	198				
Abbas	2023	*Journal of Orthopaedic Surgery and Research*	Canada	2016–2020	Economic modelling study/financial analysis	AS	V	198	198				
Blankenburg	2024	*BMC Musculoskeletal Disorders*	Germany	NR	Prospective feasibility study	Nano	IV	7	7	42.4 ± 12.8	57.1		
Blankenburg	2024	*BMC Musculoskeletal Disorders*	Germany	NR	Prospective feasibility study	AS	IV	7	7	42.4 ± 12.8	57.1		
Chowdhury	2023	*The Orthopaedic Journal of Sports Medicine*	UK	NR	Cohort study	Nano	II	22	22	(16–84)	31.8		
Chowdhury	2023	*The Orthopaedic Journal of Sports Medicine*	UK	NR	Cohort study	AS	II	22	22	(16–84)	31.8		
Deirmengian	2018	*The American Journal of Orthopedics*	USA	2015–2016	Prospective multicenter observational study	Nano	II	106	106	47 (18–82)	50		
Deirmengian	2018	*The American Journal of Orthopedics*	USA	2015–2016	Prospective multicenter observational study	AS	II	106	106	47 (18–82)	50		
Lavender	2026	*Journal of Orthopaedics*	USA	2023	Randomized controlled trial	Nano	I	41	41	48.5 ± 10.7	36.6	30.8 ± 4.9	3 (1–3)
Lavender	2026	*Journal of Orthopaedics*	USA	2023	Randomized controlled trial	AS	I	40	40	48.2 ± 9.9	52.5	32.6 ± 6.6	3 (1–3)
Munn	2023	*The Knee*	UK	2021–2022	Service feasibility/economic comparative study	Nano	IV	20	20	55.5 (32–65)	40		1
Munn	2023	*The Knee*	UK	2021–2022	Service feasibility/economic comparative study	AS	IV	20	20	55.5 (32–65)	40		1
Nakasa	2023	*Cureus*	Japan	2017–2019	Retrospective comparative cohort study	Nano	II	40	40	47.6 ± 15.6	42.5		
Nakasa	2023	*Cureus*	Japan	2017–2019	Retrospective comparative cohort study	AS	II	40	40	47.6 ± 15.6	42.5		
Schaver	2023	*Journal of Orthopaedics*	USA	2021–2022	Retrospective comparative cohort	Nano	III	19	19	42.3 ± 8.4	53	31.4 ± 5.6	1.5 (0.5–1.5)
Schaver	2023	*Journal of Orthopaedics*	USA	2021–2022	Retrospective comparative cohort	AS	III	19	19	47.6 ± 10.5	58	35.1 ± 5.4	1.5 (0.5–1.5)
Wagner	2021	*Arthroscopy*	USA	2018	Prospective clinical trial	Nano	II	50	50	47.6 (18–75)	58	26.8 (19–45)	1.5
Wagner	2021	*Arthroscopy*	USA	2018	Prospective clinical trial	AS	II	50	50	47.6 (18–75)	58	26.8 (19–45)	1.5

*Note*: The table summarizes study design, population characteristics and baseline demographics for Nano and AS. Across the nine included studies, a total of 503 patients were analysed, with comparable age and sex distributions between groups. Most studies were non‐randomized, with levels of evidence ranging from I to V, and predominantly focused on knee pathology.

Abbreviations: AS, conventional arthroscopy; BMI, body mass index; LoE, level of evidence; Nano, Needle (Nano) arthroscopy; NR, not reported; SD, standard deviation.

With regard to procedural characteristics, Needle arthroscopy was predominantly performed using commercially available Needle arthroscopy systems with scope diameters between 0.95 and 1.9 mm and cannula diameters ranging from 1.32 to 2.7 mm. Conventional arthroscopy typically employed standard 4–4.5 mm arthroscopes. Needle arthroscopy procedures were more frequently performed in office‐based settings and under local anaesthesia, whereas conventional arthroscopy was generally performed in the operating room under general anaesthesia. Device systems varied across studies and included multiple commercially available platforms, while conventional arthroscopy was performed using standard arthroscopic systems (Table 2).

**Table 2:** Technical and procedural characteristics of Needle arthroscopy and conventional arthroscopy.

First author	Procedure	Total patients, *N*	Joint	Primary indication	Device/system	Manufacturer	Scope diameter (mm)	Cannula diameter (mm)	Optic angle (°)	Field of view	Chip‐on‐tip yes/no	Local anaesthesia used yes/no	General anaesthesia used yes/no	Office setting yes/no	OR setting yes/no	Working portal yes/no
Abbas	Nano	198	Knee	Irreparable medial meniscus tear on MRI	NanoScope	Arthrex								1	1	
Abbas	AS	198	Knee	Irreparable medial meniscus tear on MRI	Standard Arthroscope										1	
Blankenburg	Nano	7	Knee	Elective knee arthroscopy/feasibility of visualization of predefined anatomical structures	NanoScope	Arthrex	1.9	2.2	0	120	1	0	1	0	1	1
Blankenburg	AS	7	Knee	Elective knee arthroscopy/feasibility of visualization of predefined anatomical structures	Standard Arthroscope	Arthrex			30			0	1	0	1	1
Chowdhury	Nano	22	Shoulder	Intra‐articular glenohumeral pathology/patients scheduled for therapeutic arthroscopy	Mi‐eye2	Trice Medical		2.1	0			0	1	0	1	0
Chowdhury	AS	22	Shoulder	Intra‐articular glenohumeral pathology/patients scheduled for therapeutic arthroscopy	Standard Arthroscope		4		30			0	1	0	1	0
Deirmengian	Nano	106	Knee	Planned arthroscopic knee procedure based on history, physical exam and MRI findings	Mi‐eye+	Trice Medical		2.1			1	0	1	0	1	1
Deirmengian	AS	106	Knee	Planned arthroscopic knee procedure based on history, physical exam and MRI findings	Standard Arthroscope						1	0	1	0	1	1
Lavender	Nano	41	Knee	Isolated partial meniscectomy	Nanoscope	Arthrex	1.9				1	0	1	0	1	1
Lavender	As	40	Knee	Isolated partial meniscectomy	Standard Arthroscope						1	0	1	0	1	1
Munn	Nano	20	Knee	Refractory knee symptoms/chronic soft tissue or early degenerative disease	Nanoscope	Arthrex		2.7			1	1	0	1	0	1
Munn	As	20	Knee	Refractory knee symptoms/chronic soft tissue or early degenerative disease	Standard Arthroscope	Stryker							1	0	1	
Nakasa	Nano	40	Knee	Suspected meniscal injury	Needle arthroscope	Smith & Nephew	0.95	1.32	115			0	1	0	1	1
Nakasa	As	40	Knee	Suspected meniscal injury	Standard Arthroscope		4.5						1			
Schaver	Nano	19	Knee	Partial meniscectomy/meniscus tear	NanoScope	Arthrex	1.9		0	120	1	0	1	0	1	1
Schaver	As	19	Knee	Partial meniscectomy/meniscus tear	Standard Arthroscope				0			0	1	0	1	1
Wagner	Nano	50	Shoulder	Suspected intra‐articular shoulder pathology requiring arthroscopy	Mi‐Eye	Trice Medical		2.1	0		1	0	1	0	1	1
Wagner	As	50	Shoulder	Suspected intra‐articular shoulder pathology requiring arthroscopy	Standard Arthroscope								1	0	1	

*Note*: The table provides a detailed overview of devices, procedural settings and technical parameters. Needle arthroscopy systems were characterized by smaller scope and cannula diameters and were more frequently used in office‐based settings under local anaesthesia, whereas conventional arthroscopy was predominantly performed in the operating room under general anaesthesia. Variability in device type, optical configuration and clinical indication was observed across studies.

Abbreviations: AS, conventional arthroscopy; MRI, magnetic resonance imaging; Nano, Needle (Nano) arthroscopy; NR, not reported; OR, operating room.

### Risk of bias assessment

A total of nine studies were assessed for risk of bias, including eight non‐randomized studies [1, 9, 14, 19, 55, 61, 75, 88] evaluated using the ROBINS‐I tool and one RCT [45] assessed using RoB 2 (Table 3). Among the non‐randomized studies, two studies [1, 55] were judged to be at critical risk of bias, primarily due to their non‐comparative or model‐based design, which precluded adequate control for confounding and introduced substantial limitations in outcome measurement. Two studies [9, 75] were rated as having a serious risk of bias, mainly driven by participant selection, small sample sizes and non‐randomized study designs with potential confounding. The remaining four non‐randomized studies [14, 19, 61, 88] were judged to be at moderate risk of bias. Although these studies generally applied consistent reference standards and, in some cases, blinded outcome assessment, they were limited by selective inclusion of surgical populations, lack of randomization and potential measurement bias. The only RCT [45] demonstrated some concerns according to the RoB 2. While the randomization process and completeness of outcome data were adequate, potential bias arose from the lack of blinding of surgeons and the use of partly subjective outcome measures. Overall, the body of evidence [1, 9, 14, 19, 45, 55, 61, 75, 88] was characterized by predominantly moderate to high risk of bias, with only one randomized study and no study achieving a low overall risk of bias (Table 3).

**Table 3:** Risk of bias assessment of included studies.

Study	Tool	Confounding	Selection	Classification	Deviations	Missing data	Outcome measurement	Reporting	Overall
Abbas 2023	ROBINS‐I	Critical	Critical	Serious	NA	Low	Serious	Serious	Critical
Blankenburg 2024	ROBINS‐I	Moderate	Serious	Low	Moderate	Low	Moderate	Moderate	Serious
Chowdhury 2023	ROBINS‐I	Moderate	Moderate	Low	Low	Low	Moderate	Low	Moderate
Deirmengian 2018	ROBINS‐I	Moderate	Moderate	Low	Low	Moderate	Moderate–Serious	Low	Moderate
Munn 2023	ROBINS‐I	Critical	Critical	Moderate	NA	Low	Serious	Serious	Critical
Nakasa 2023	ROBINS‐I	Moderate	Serious	Low	Low	Low	Moderate	Moderate	Moderate
Schaver 2023	ROBINS‐I	Serious	Moderate	Low	Moderate	Moderate	Moderate	Moderate	Serious
Wagner 2021	ROBINS‐I	Moderate	Moderate	Low	Low	Low	Moderate	Low	Moderate
Lavender 2026	RoB 2	Low*	Low*	—	Some concerns	Low	Some concerns	Some concerns	Some concerns

*Note*: Non‐randomized studies were evaluated using the ROBINS‐I tool and the randomized controlled trial using RoB 2. Most studies were judged to be at moderate to critical risk of bias, primarily due to confounding, participant selection and lack of randomization.

Abbreviations: NA, not applicable; RoB 2, Risk of Bias 2 tool; ROBINS‐I, Risk Of Bias In Non‐randomized Studies of Interventions.

* Low risk of bias according to the RoB 2 assessment; the corresponding ROBINS‐I domains are not directly applicable.

### Certainty of evidence

The certainty of evidence ranged from moderate to very low across outcomes (Table 4). Procedural outcomes, including procedure completion and successful diagnosis, were rated as moderate certainty, reflecting consistent results and low heterogeneity despite non‐randomized designs. Clinical outcomes were supported by low certainty evidence. KOOS4 at 0.5 months showed a significant benefit for needle arthroscopy, whereas KOOS4 at 1.5 months did not, with both outcomes limited by small study numbers and imprecision. Safety outcomes were also rated as low certainty, with very low certainty for rare events such as infection and reoperation due to sparse data and wide CIs. Management change was rated as low certainty for similar reasons. All diagnostic accuracy outcomes were rated as very low certainty, due to high risk of bias, heterogeneity, indirectness and reconstructed data. Overall, no outcome reached high certainty of evidence (Table 4).

**Table 4:** Certainty of evidence assessed using the GRADE approach.

Outcome	Effect estimate	Studies	Risk of bias	Inconsistency	Indirectness	Imprecision
Procedure completed	OR 1.00 (0.17–5.93)	5	Serious ↓	Not serious	Not serious	Not serious
Successful diagnosis	OR 1.01 (0.10–10.07)	3	Serious ↓	Not serious	Not serious	Not serious
Any complication	OR 1.00 (0.20–5.06)	5	Serious ↓	Not serious	Not serious	Serious ↓
Minor complication	OR 1.36 (0.25–7.41)	5	Serious ↓	Not serious	Not serious	Serious ↓
Infection	OR 1.00 (0.17–5.88)	5	Serious ↓	Not serious	Not serious	Very serious ↓↓
Reoperation	OR 1.29 (0.27–6.11)	6	Serious ↓	Not serious	Not serious	Very serious ↓↓
Management change	OR 1.47 (0.22–9.63)	4	Serious ↓	Not serious	Not serious	Serious ↓
TP	OR 0.48 (0.07–3.01)	2	Serious ↓	Serious ↓	Serious ↓	Serious ↓
TN	OR 0.42 (0.04–4.44)	2	Serious ↓	Serious ↓	Serious ↓	Serious ↓
FN	OR 5.82 (0.66–51.43)	2	Serious ↓	Serious ↓	Serious ↓	Serious ↓
FP	OR 5.06 (0.47–54.60)	2	Serious ↓	Serious ↓	Serious ↓	Serious ↓
Sensitivity	OR 0.29 (0.04–2.33)	3	Serious ↓	Serious ↓	Serious ↓	Serious ↓
Specificity	OR 0.71 (0.05–9.88)	3	Serious ↓	Serious ↓	Serious ↓	Serious ↓
Baseline KOOS4	MD 2.51 (−4.50 to 9.52)	2	Serious ↓	Not serious	Not serious	Serious ↓
KOOS4 (0.5 months)	MD 15.25 (7.82–22.67)	2	Serious ↓	Not serious	Not serious	Serious ↓
KOOS4 (1.5 months)	MD 7.25 (0.27–14.22)	2	Serious ↓	Not serious	Not serious	Serious ↓

*Note*: The certainty of evidence ranged from moderate for procedural outcomes to low or very low for clinical, safety and diagnostic accuracy outcomes, mainly due to risk of bias, imprecision and heterogeneity.

Abbreviations: CI, confidence interval; FN, false negative; FP, false positive; GRADE, Grading of Recommendations Assessment, Development and Evaluation; KOOS, Knee injury and Osteoarthritis Outcome Score; KOOS4, KOOS composite score (mean of four subscales); MD, mean difference; OR, odds ratio; TN, true negative; TP, true positive.

### Assessment of publication bias

Funnel plots were generated for outcomes with sufficient data (Figures [Link], [Link], [Link], [Link], [Link], [Link], [Link], [Link], [Link], [Link], [Link], [Link], [Link], [Link], [Link], [Link]). Visual inspection of funnel plots for procedure completion, successful diagnosis, complications and KOOS4 outcomes did not reveal clear asymmetry, suggesting no obvious small‐study effects. However, the interpretability of these plots was limited by the small number of contributing studies per outcome and by the presence of zero‐event data for several endpoints. For diagnostic accuracy outcomes, including sensitivity and specificity, the funnel plots showed greater dispersion, consistent with substantial between‐study heterogeneity rather than clear evidence of publication bias. Overall, due to the limited number of studies per outcome, funnel plot assessments should be interpreted cautiously.

### Meta‐analysis

#### Procedural and clinical outcomes

**Figure 2:**
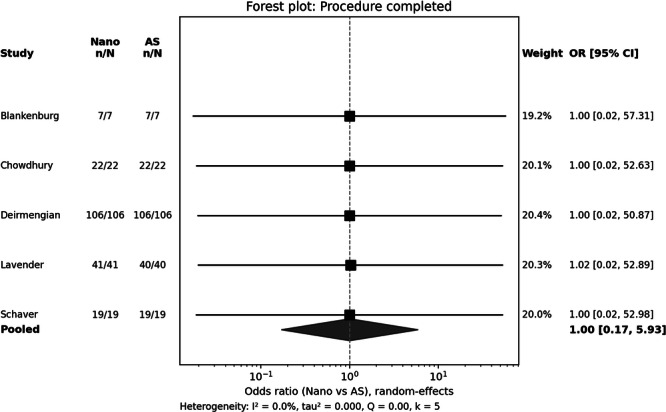
Forest plot comparing Nano and AS for procedure completion rate. Procedure completion rates were high in both groups without a significant difference (OR 1.00, 95% CI 0.17–5.93; *I* ^2^ = 0.0%). AS, conventional arthroscopy; CI, confidence interval; Nano, Needle (Nano) arthroscopy; OR, odds ratio.

Procedure completion rate: Five studies [9, 14, 19, 45, 75] contributed data. Procedure completion rates were comparable between needle arthroscopy and conventional arthroscopy (OR 1.00, 95% CI 0.17–5.93; *I* ^2 ^= 0%) (Figure 2, Table 5).

**Table 5:** Summary of meta‐analysis results comparing Needle arthroscopy and conventional arthroscopy.

Endpoint	Type	Studies	Effect	LCL	UCL	*I* ^2^%	*τ* ^2^	*Q*
Procedure completed	Binary (OR)	5	1.00	0.17	5.93	0.00	0.00	0.00
Successful diagnosis	Binary (OR)	3	1.01	0.10	10.07	0.00	0.00	0.00
Any complication	Binary (OR)	5	1.00	0.20	5.06	0.00	0.00	0.95
Minor complication	Binary (OR)	5	1.36	0.25	7.41	0.00	0.00	0.35
Infection	Binary (OR)	5	1.00	0.17	5.88	0.00	0.00	0.00
Reoperation	Binary (OR)	6	1.29	0.27	6.11	0.00	0.00	0.00
Management change	Binary (OR)	4	1.47	0.22	9.63	0.00	0.00	0.00
TP	Binary (OR)	2	0.48	0.07	3.01	45.90	1.04	1.85
TN	Binary (OR)	2	0.42	0.04	4.44	62.40	2.00	2.66
FN	Binary (OR)	2	5.82	0.66	51.43	0.00	0.00	0.28
FP	Binary (OR)	2	5.06	0.47	54.60	2.00	0.06	1.02
Sensitivity	Binary (OR)	3	0.29	0.04	2.33	58.2	1.97	4.78
Specificity	Binary (OR)	3	0.71	0.05	9.88	47.90	2.59	3.84
Baseline KOOS4	Continuous (MD)	2	2.51	−4.50	9.52	0.00	0.00	0.74
KOOS4 at 0.5 months	Continuous (MD)	2	15.25	7.82	22.67	0.00	0.00	0.97
KOOS4 at 1.5 months	Continuous (MD)	2	7.25	0.27	14.22	0.00	0.00	0.58

*Note*: Effect estimates are presented as OR for dichotomous outcomes and MDs for continuous outcomes with corresponding 95% CIs. Most outcomes showed no significant differences between groups, except for a short‐term functional benefit in KOOS4 at 0.5 months.

Abbreviations: CI, confidence interval; FN, false negative; FP, false positive; *I* ^2^, proportion of the total variation across studies that is due to true heterogeneity rather than chance; KOOS, Knee injury and Osteoarthritis Outcome Score; KOOS4, KOOS composite score (mean of four subscales); LCL, lower confidence limit; MD, mean difference; OR, odds ratio; *Q* (Cochran's Q), statistical test for heterogeneity assessing whether observed differences between studies exceed what would be expected by chance; TN, true negative; TP, true positive; UCL, upper confidence limit; *τ* ^2^, estimate of the between‐study variance, reflecting the dispersion of true effect sizes.

**Figure 3:**
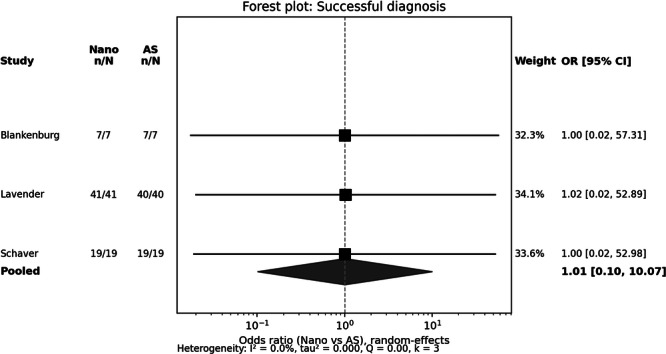
Forest plot comparing Nano and AS for successful diagnosis. Diagnostic success rates exceeded 90% in both groups, with no significant difference observed (OR 1.01, 95% CI 0.10–10.07; *I* ^2 ^= 0.0%). AS, conventional arthroscopy; CI, confidence interval; Nano, Needle (Nano) arthroscopy; OR, odds ratio.

Successful diagnosis: Three studies [9, 45, 75] contributed to the analysis. Diagnostic success rates were similar between groups (OR 1.01, 95% CI 0.10–10.07; *I* ^2 ^= 0%) (Figure 3, Table 5).

**Figure 4:**
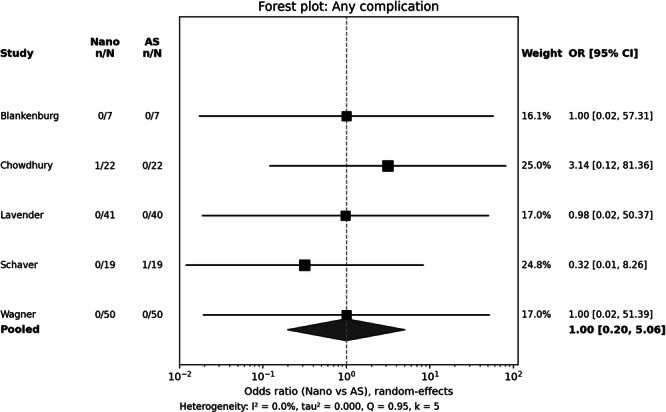
Forest plot comparing Nano and AS for any complication. Overall complication rates were low and comparable between groups (OR 1.00, 95% CI 0.20–5.06; *I* ^2 ^= 0.0%). AS, conventional arthroscopy; CI, confidence interval; Nano, Needle (Nano) arthroscopy; OR, odds ratio.

Any complication: Five studies [9, 14, 45, 75, 88] were included. Overall complication rates were low and did not differ significantly between Needle and conventional arthroscopy (OR 1.00, 95% CI 0.20–5.06; *I* ^2 ^= 0%) (Figure 4, Table 5).

**Figure 5:**
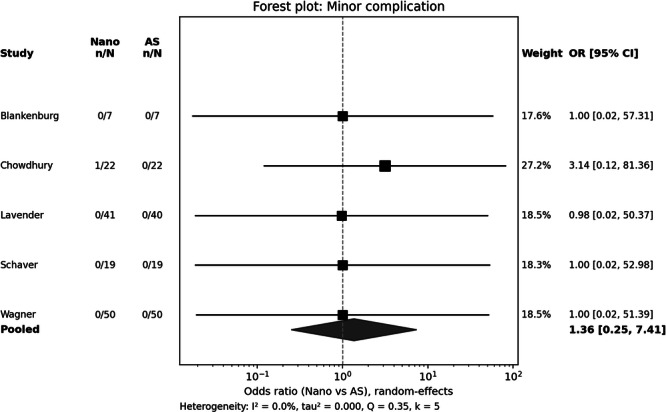
Forest plot comparing Nano and AS for minor complications. Minor complications were infrequent and did not differ significantly between techniques (OR 1.36, 95% CI 0.25–7.41; *I* ^2 ^= 0.0%). AS, conventional arthroscopy; CI, confidence interval; Nano, Needle (Nano) arthroscopy; OR, odds ratio.

Minor complication: Five studies [9, 14, 45, 75, 88] contributed data. Minor complications were infrequent, with no significant difference between groups (OR 1.36, 95% CI 0.25–7.41; *I* ^2 ^= 0%) (Figure 5, Table 5). Reported complications were rare and included one iatrogenic partial‐thickness humeral head cartilage injury during needle arthroscope insertion [14] and one postoperative deep vein thrombosis in the conventional arthroscopy group [75].

**Figure 6:**
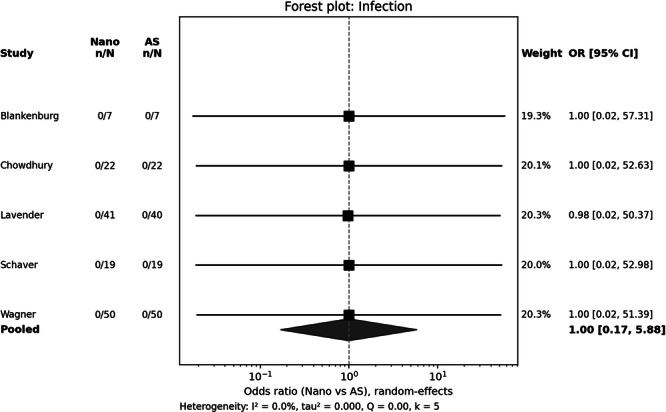
Forest plot comparing Nano and AS for infection rates. Infection events were rare, with no difference observed between groups (OR 1.00, 95% CI 0.17–5.88; *I* ^2 ^= 0.0%). AS, conventional arthroscopy; CI, confidence interval; Nano, Needle (Nano) arthroscopy; OR, odds ratio.

Infection: Five studies [9, 14, 45, 75, 88] reported infection outcomes. Infection rates were rare, with no difference observed between groups (OR 1.00, 95% CI 0.17–5.88; *I* ^2 ^= 0%) (Figure 6, Table 5).

**Figure 7:**
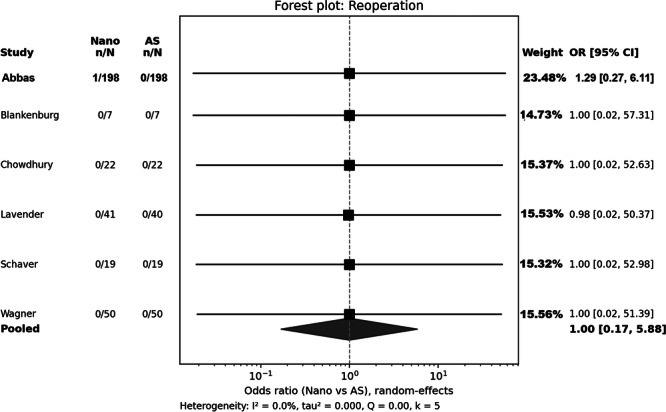
Forest plot comparing Nano and AS for reoperation. Reoperation rates were very low, with no meaningful difference between groups and wide CIs indicating imprecision (OR 1.29, 95% CI 0.27–6.11; *I* ^2 ^= 0.0%). AS, conventional arthroscopy; CI, confidence interval; Nano, Needle (Nano) arthroscopy; OR, odds ratio.

Reoperation: Six studies [1, 9, 14, 45, 75, 88] contributed to this outcome. Reoperation rates were very low and did not differ between groups (OR 1.29, 95% CI 0.27–6.11; *I* ^2^ = 0%) (Figure 7, Table 5).

**Figure 8:**
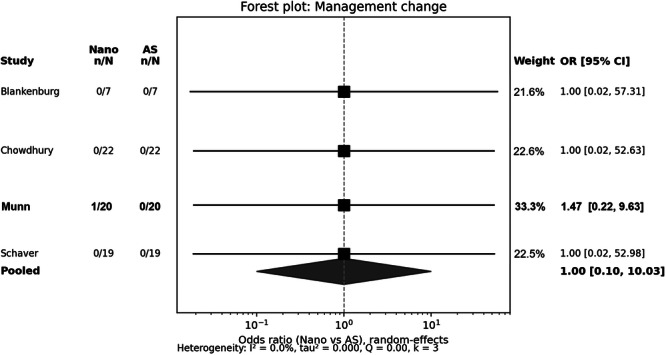
Forest plot comparing Nano and AS for management change. The rate of management changes was low and comparable between groups (OR 1.47, 95% CI 0.22–9.63; *I* ^2 ^= 0.0%). AS, conventional arthroscopy; CI, confidence interval; Nano, Needle (Nano) arthroscopy; OR, odds ratio.

Management change: Four studies [9, 14, 61, 75] reported management change outcomes. No significant difference was found between groups (OR 1.47, 95% CI 0.22–9.63; *I* ^2 ^= 0%) (Figure 8, Table 5).

### Diagnostic performance outcomes

**Figure 9:**
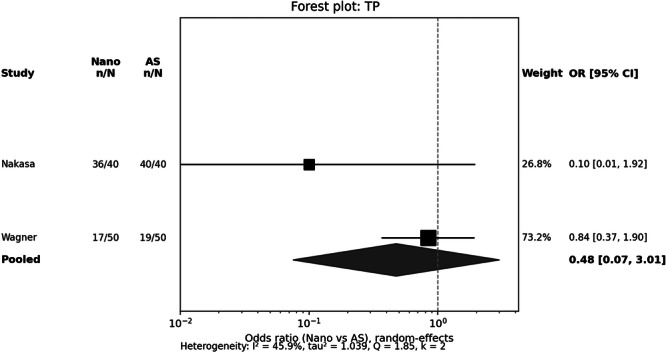
Forest plot comparing Nano and AS for true positives (TPs). TP rates were high in both groups, with no significant difference observed and considerable variability across studies (OR 0.48, 95% CI 0.07–3.01; *I* ^2 ^= 45.9%). AS, conventional arthroscopy; CI, confidence interval; Nano, Needle (Nano) arthroscopy; OR, odds ratio.

True positives (TPs): Two studies [61, 88] contributed data. The proportion of TP findings did not differ significantly between groups (OR 0.48, 95% CI 0.07–3.01; *I* ^2 ^= 45.9%) (Figure 9, Table 5).

**Figure 10:**
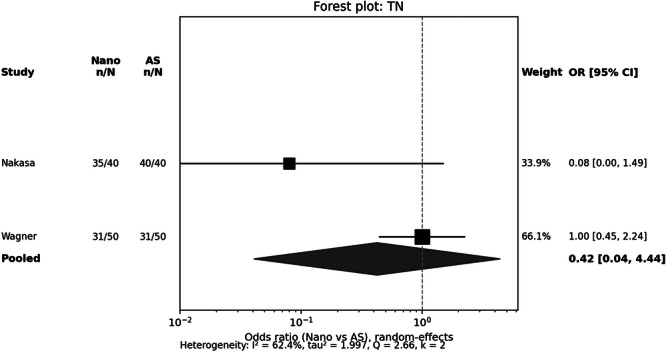
Forest plot comparing Nano and AS for true negatives (TNs). TN rates were similarly high and did not differ significantly between techniques (OR 0.42, 95% CI 0.04–4.44; *I* ^2 ^= 62.4%). AS, conventional arthroscopy; CI, confidence interval; Nano, Needle (Nano) arthroscopy; OR, odds ratio.

True negatives (TNs): Two studies [61, 88] contributed data. TN rates were comparable between techniques (OR 0.42, 95% CI 0.04–4.44; *I* ^2 ^= 62.4%) (Figure 10, Table 5).

**Figure 11:**
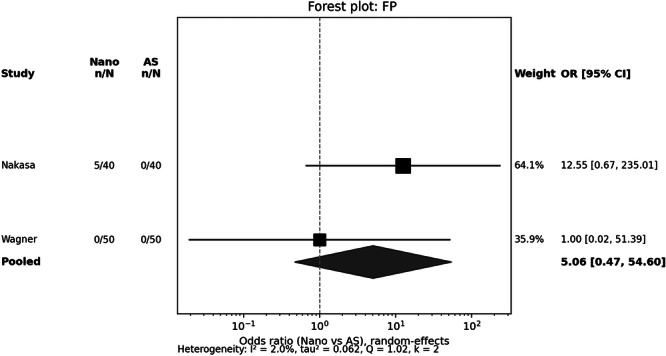
Forest plot comparing Nano and AS for false positives (FPs). FP rates were low across studies, with no significant difference between groups (OR 5.06, 95% CI 0.47–54.60; *I* ^2 ^= 2.0%). AS, conventional arthroscopy; CI, confidence interval; Nano, Needle (Nano) arthroscopy; OR, odds ratio.

False positives (FPs): Two studies [61, 88] reported FPs. Rates did not differ significantly between techniques (OR 5.06, 95% CI 0.47–54.60; *I* ^2 ^= 2.0%) (Figure 11, Table 5).

**Figure 12:**
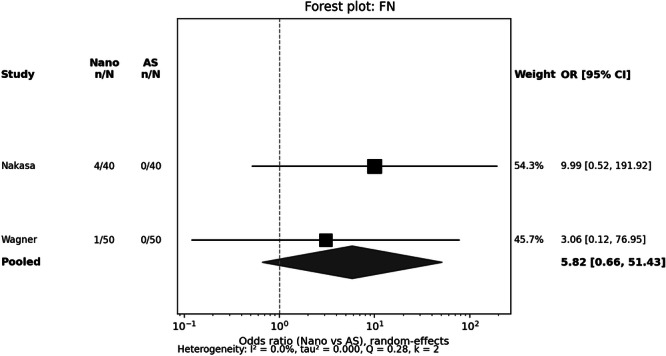
Forest plot comparing Nano and AS for false negatives (FNs). FN rates were low and comparable between techniques, although estimates were imprecise (OR 5.82, 95% CI 0.66–51.43; *I* ^2 ^= 0.0%). AS, conventional arthroscopy; CI, confidence interval; Nano, Needle (Nano) arthroscopy; OR, odds ratio.

False negatives (FNs): Two studies [61, 88] reported FNs. No significant difference was observed between groups (OR 5.82, 95% CI 0.66–51.43; *I* ^2 ^= 0%) (Figure 12, Table 5).

**Figure 13:**
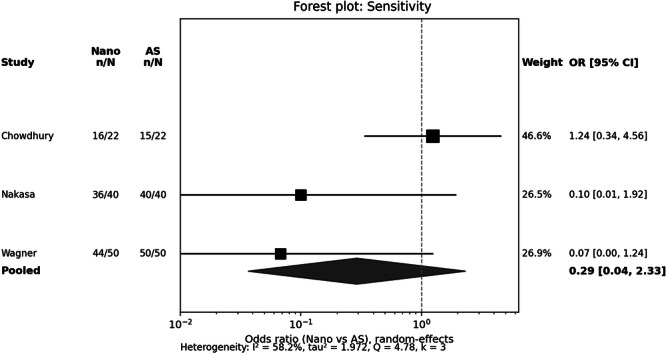
Forest plot comparing Nano and AS for sensitivity. No significant difference in sensitivity was observed, with substantial heterogeneity across studies (OR 0.29, 95% CI 0.04–2.33; *I* ^2 ^= 58.2%). AS, conventional arthroscopy; CI, confidence interval; Nano, Needle (Nano) arthroscopy; OR, odds ratio.

Sensitivity: Three studies [14, 61, 88] contributed to the analysis. Sensitivity did not differ significantly between Needle and conventional arthroscopy (OR 0.29, 95% CI 0.04–2.33; *I* ^2 ^= 58.2%) (Figure 13, Table 5).

**Figure 14:**
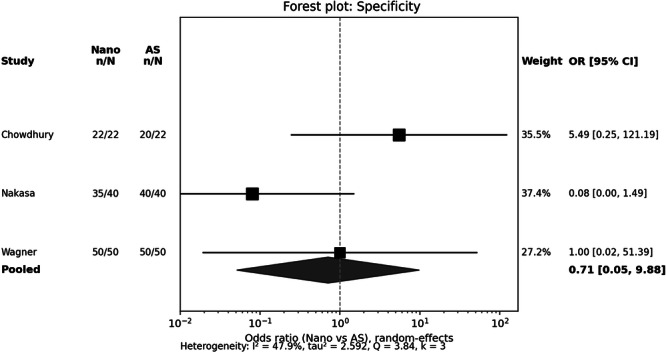
Forest plot comparing Nano and AS for specificity. Specificity did not differ significantly between groups, with high heterogeneity and wide CIs (OR 0.71, 95% CI 0.05–9.88; *I* ^2 ^= 47.9%). AS, conventional arthroscopy; CI, confidence interval; Nano, Needle (Nano) arthroscopy; OR, odds ratio.

Specificity: Three studies [14, 61, 88] were included. No significant difference in specificity was observed between techniques (OR 0.71, 95% CI 0.05–9.88; *I* ^2 ^= 47.9%) (Figure 14, Table 5).

### Patient‐reported outcomes

**Figure 15:**
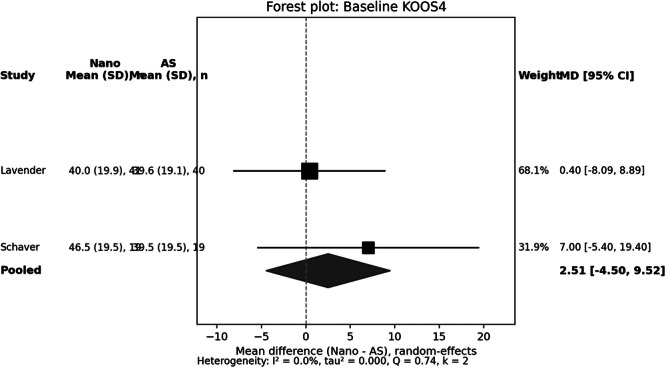
Forest plot comparing Nano and AS for KOOS4 at baseline. Baseline KOOS4 scores were comparable between groups, indicating similar pre‐interventional functional status (MD 2.51, 95% CI −4.50 to 9.52; *I* ^2 ^= 0.0%). AS, conventional arthroscopy; CI, confidence interval; KOOS, Knee injury and Osteoarthritis Outcome Score; KOOS4, KOOS composite score (mean of four subscales); MD, mean difference; Nano, Needle (Nano) arthroscopy; SD, standard deviation.

KOOS4 (baseline): Two studies [45, 75] contributed to the baseline analysis. There was no significant difference in baseline KOOS4 scores between groups (MD 2.51, 95% CI −4.50 to 9.52; *I* ^2 ^= 0%) (Figure 15, Table 5).

**Figure 16:**
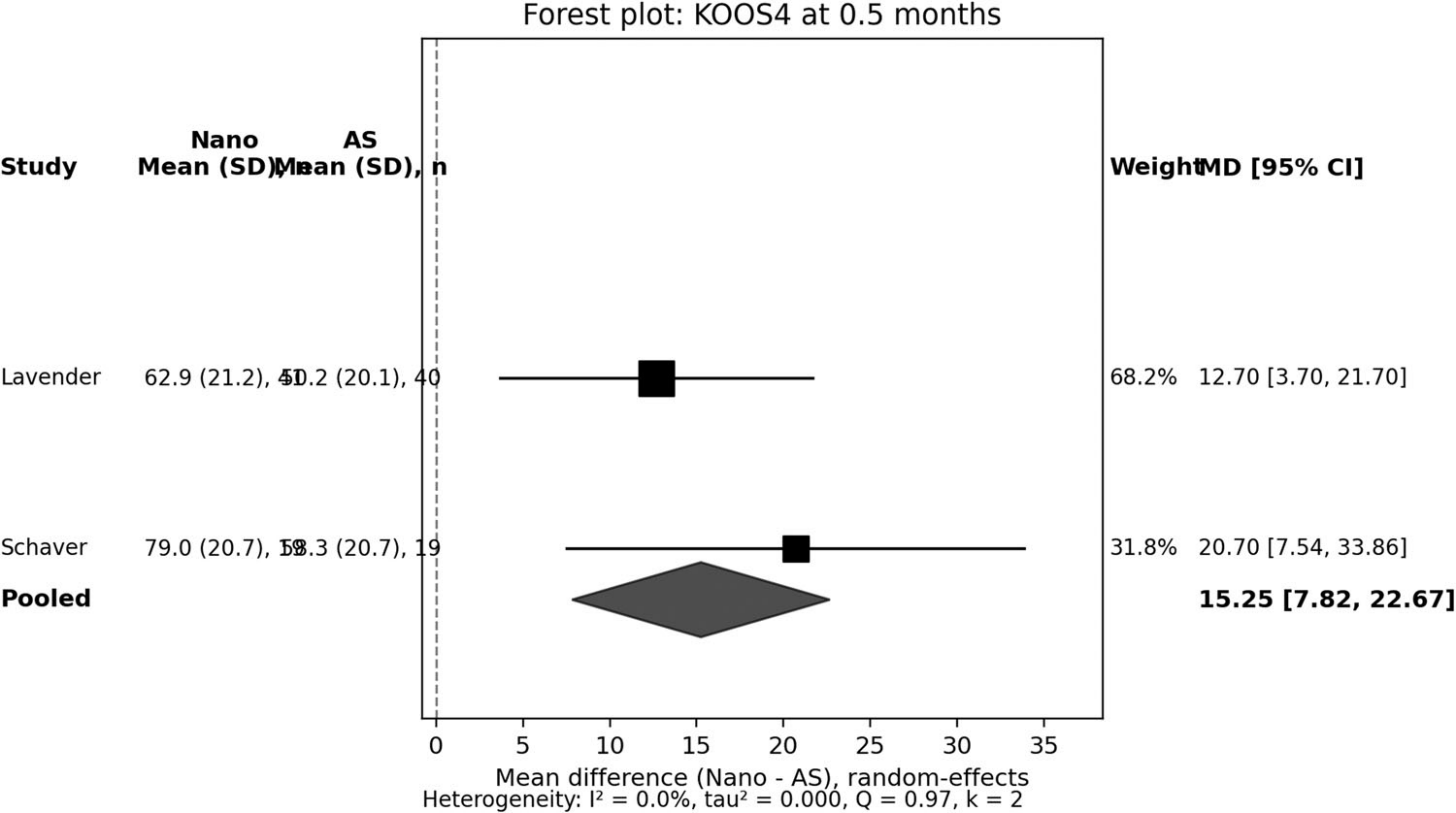
Forest plot comparing Nano and AS for KOOS4 at 0.5 months. Needle arthroscopy showed a significant improvement in early functional outcome (MD 15.25, 95% CI 7.82–22.67; *I* ^2 ^= 0.0%). AS, conventional arthroscopy; CI, confidence interval; KOOS, Knee injury and Osteoarthritis Outcome Score; KOOS4, KOOS composite score (mean of four subscales); MD, mean difference; Nano, Needle (Nano) arthroscopy; SD, standard deviation.

KOOS4 at 0.5 months: Two studies [45, 75] reported short‐term outcomes. Needle arthroscopy was associated with significantly higher KOOS4 scores at 0.5 months (MD 15.25, 95% CI 7.82–22.67; *I* ^2 ^= 0%) (Figure 16, Table 5).

**Figure 17:**
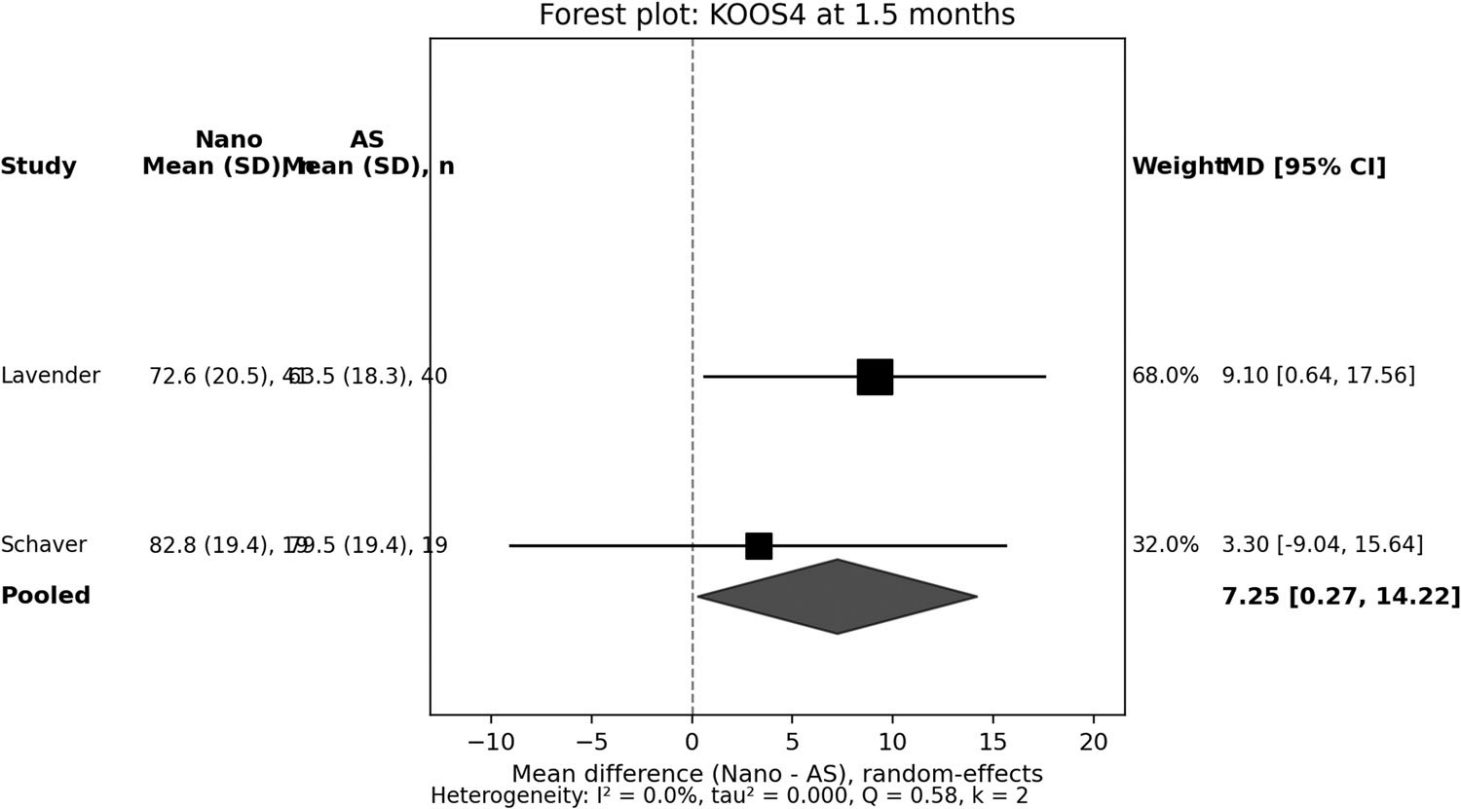
Forest plot comparing Nano and AS for KOOS4 at 1.5 months. The difference between groups decreased but remained statistically significant (MD 7.25, 95% CI 0.27–14.22; *I* ^2 ^= 0.0%). AS, conventional arthroscopy; CI, confidence interval; KOOS, Knee injury and Osteoarthritis Outcome Score; KOOS4, KOOS composite score (mean of four subscales); MD, mean difference; Nano, Needle (Nano) arthroscopy; SD, standard deviation.

KOOS4 at 1.5 months: Two studies [45, 75] contributed data at 1.5 months. KOOS4 remained higher in the needle arthroscopy group (MD 7.25, 95% CI 0.27–14.22; *I* ^2 ^= 0%) (Figure 17, Table 5).

## DISCUSSION

### Summary of findings

This meta‐analysis compared Needle arthroscopy with conventional arthroscopy across functional, procedural and diagnostic outcomes. The main finding is that Needle arthroscopy demonstrated promising diagnostic performance and short‐ to mid‐term functional advantages without an increase in complication rates. Overall, the available evidence suggests that Needle arthroscopy may represent a valuable adjunct or emerging minimally invasive option in selected clinical settings.

### Functional outcomes

With regard to functional outcomes, Needle arthroscopy was associated with a statistically significant improvement in KOOS4 scores at 0.5 months (MD +15.25 points, 95% CI 7.82–22.67), indicating superior early recovery. This advantage remained statistically significant at 1.5 months (MD +7.25 points, 95% CI 0.27–14.22), although with a reduced effect size. The likely mechanism relates to the minimally invasive nature of Needle arthroscopy, which avoids formal surgical portals and reduces soft‐tissue disruption.

### Diagnostic and procedural performance

Both techniques demonstrated high procedural success and diagnostic performance across the included studies. Procedure completion and successful diagnosis rates were comparable between groups, and the frequency of subsequent management changes did not differ significantly. These findings suggest that Needle arthroscopy may provide sufficient diagnostic information for clinical decision‐making in selected patients without compromising technical feasibility. Beyond this formal comparability, one of the main potential advantages of Needle arthroscopy lies in its integration into office‐based care pathways. In appropriately selected patients, it may accelerate diagnosis and treatment decisions, avoid general anaesthesia and improve convenience and tolerability under local anaesthesia. In addition, emerging evidence suggests that patients may prefer IONA over traditional surgical arthroscopy and that, in selected scenarios, it may reduce operating‐room utilization and potentially lower overall resource use [1, 55, 73].

### Complications, prevention and clinical management

Overall complication rates were low and did not differ significantly between Needle arthroscopy and conventional arthroscopy, with no significant differences in overall complications, minor complications, infection or reoperation rates. Reported adverse events were rare and limited to one iatrogenic partial‐thickness humeral head cartilage injury during Needle arthroscope insertion in the Needle arthroscopy group and one postoperative deep vein thrombosis in the conventional arthroscopy group. Although interpretation is limited by multiple zero‐event studies and small sample sizes, no safety signal favouring or opposing either technique was identified. From a clinical perspective, careful patient selection, adequate training, familiarity with the instrumentation and meticulous portal placement are likely to minimize procedure‐related complications. Appropriate preoperative planning and adherence to established arthroscopic principles remain essential, while the reported adverse events can generally be managed according to standard arthroscopic practice. Overall, the available evidence supports the safety of Needle arthroscopy when performed by appropriately trained surgeons in selected patients.

### Diagnostic accuracy

Exploratory analyses of diagnostic accuracy showed no significant differences in sensitivity or specificity between the two techniques, although substantial heterogeneity was observed. These findings must be interpreted with caution, as diagnostic data were inconsistently reported, frequently lacked complete 2 × 2 contingency tables and often required reconstruction from reported percentages. In addition, in several studies conventional arthroscopy served as the reference standard, inherently favouring its apparent performance. Consequently, no definitive conclusions regarding diagnostic superiority can be drawn.

### Comparison with previous literature

Our findings are consistent with prior systematic reviews while providing a more rigorous quantitative synthesis. Previous reviews reported high diagnostic accuracy and potential advantages of Needle arthroscopy over imaging modalities for selected intra‐articular pathologies [10, 51, 89, 96]. More recent prospective investigations further demonstrated that small‐bore Needle arthroscopy may achieve diagnostic performance comparable or even superior to MRI for selected shoulder pathologies, particularly involving the rotator cuff and long head of the biceps [11]. Furthermore, emerging evidence suggests expanding therapeutic applications of Needle arthroscopy beyond purely diagnostic indications, including fracture‐assisted reduction techniques, hindfoot endoscopy and minimally invasive ankle procedures [56, 76, 93]. However, previous reviews and most primary investigations were largely descriptive and did not include quantitative benchmarking or pooled comparative estimates against conventional arthroscopy. In contrast, the present study suggests at the meta‐analytic level that Needle arthroscopy may achieve comparable diagnostic and procedural performance while offering improved short‐term functional outcomes.

Similarly, earlier reviews have highlighted the broad clinical applicability of Needle arthroscopy but emphasized the limited methodological quality of the available evidence. This review corroborates these concerns, as most included studies were non‐randomized and exhibited moderate to serious risk of bias. The application of formal meta‐analytic methods and GRADE assessment in the present analysis provides a more robust synthesis but confirms that the overall certainty of evidence remains limited.

With respect to safety, recent evidence has consistently reported low complication rates following Needle arthroscopy, with most complications being minor. Our findings align with these observations and further demonstrate that complication rates are comparable to those of conventional arthroscopy.

### Limitations and future directions

Several limitations must be acknowledged. Most included studies were non‐randomized and susceptible to confounding and selection bias. Sample sizes were small, and outcome reporting was heterogeneous, particularly for diagnostic accuracy parameters. The inclusion of both office‐based and operating‐room procedures further limits comparability. Additionally, the high prevalence of zero‐event data in complication analyses reduces statistical precision.

Future research should move beyond feasibility and short‐term comparative studies and focus on clearly defined clinical indications in which Needle arthroscopy may provide added value over conventional diagnostic pathways. In particular, office‐based Needle arthroscopy may be most relevant as a diagnostic or second‐look tool, for postoperative follow‐up, equivocal imaging findings and selected minor therapeutic procedures. Recent In‐office and Needle‐based applications have demonstrated the feasibility of direct intra‐articular visualization outside the conventional operating‐room environment, while emerging Needle arthroscopy‐assisted approaches also suggest potential for minimally invasive tissue assessment and translational research applications.

However, these developments should be interpreted cautiously. Current evidence does not yet support broad therapeutic equivalence with conventional arthroscopy, particularly when complex reconstructive or definitive therapeutic procedures are anticipated. Future studies should therefore separately evaluate diagnostic accuracy, procedural feasibility, patient‐reported outcomes, complication rates, cost‐effectiveness and therapeutic efficacy rather than combining these domains under broad terms such as ‘effectiveness’. Larger prospective multicenter studies and adequately powered RCTs are needed, with standardized definitions of procedural success, complete 2 × 2 diagnostic accuracy reporting, joint‐specific outcome measures and longer follow‐up. In addition, future work should address technical factors that may affect generalizability, including image quality, the use of 0° optics, fluid management, instrument limitations, the learning curve and the practical requirements of office‐based implementation.

Taken together, Needle arthroscopy appears to be a promising minimally invasive alternative to conventional arthroscopy, particularly in settings where early functional recovery and reduced invasiveness are prioritized. However, conventional arthroscopy remains the reference standard, particularly when therapeutic intervention is anticipated. Further high‐quality RCTs are required to confirm these findings and better define optimal indications, patient selection criteria and long‐term outcomes.

## CONCLUSION

Needle arthroscopy demonstrates promising diagnostic and procedural performance with low complication rates and potential short‐term functional advantages. However, due to the limited quality and heterogeneity of the available evidence, conventional arthroscopy remains the current reference standard, particularly for definitive therapeutic intervention. Further high‐quality comparative studies are required to better define the clinical role of needle arthroscopy.

## AUTHOR CONTRIBUTIONS

Nikolai Ramadanov and Maximilian Voss performed the literature search, the data extraction and the risk of bias assessment. Nikolai Ramadanov and Ingo J. Banke conducted the statistical calculations. Nikolai Ramadanov created all figures and tables. Ingo J. Banke wrote the manuscript. Nikolai Ramadanov, Roland Becker and Maximilian Voss supervised the work.

## FUNDING INFORMATION

The authors have no funding to report.

## CONFLICT OF INTEREST STATEMENT

The authors declare no conflicts of interest.

## ETHICS STATEMENT

This study was registered in PROSPERO on 9 March 2026 (registration number: CRD420261335973).

## Supporting information

Supplementary Figure 1: **Funnel plot comparing needle arthroscopy and conventional arthroscopy for procedure completion rate.** The distribution appears symmetrical, suggesting no relevant small‐study effects.

Supplementary Figure 2: **Funnel plot comparing needle arthroscopy and conventional arthroscopy for successful diagnosis.** No clear asymmetry was observed, indicating a low likelihood of publication bias.

Supplementary Figure 3: **Funnel plot comparing needle arthroscopy and conventional arthroscopy for any complication.** Interpretation is limited due to low event rates and a small number of studies.

Supplementary Figure 4: **Funnel plot comparing needle arthroscopy and conventional arthroscopy for minor complications.** The plot does not suggest substantial asymmetry, although conclusions are limited by sparse data.

Supplementary Figure 5: **Funnel plot comparing needle arthroscopy and conventional arthroscopy for infection rates.** Assessment is limited due to very low event counts and insufficient study numbers.

Supplementary Figure 6: **Funnel plot comparing needle arthroscopy and conventional arthroscopy for reoperation.** Interpretation is not reliable due to the small number of studies and rare events.

Supplementary Figure 7: **Funnel plot comparing needle arthroscopy and conventional arthroscopy for management change.** No clear asymmetry was observed, but the small number of studies limits interpretation.

Supplementary Figure 8: **Funnel plot comparing needle arthroscopy and conventional arthroscopy for true positives (TP).** Visual interpretation suggests variability across studies; however, results are based on reconstructed data and should be interpreted with caution.

Supplementary Figure 9: **Funnel plot comparing needle arthroscopy and conventional arthroscopy for true negatives (TN).** No clear pattern of asymmetry was observed, although precision is limited.

Supplementary Figure 10: **Funnel plot comparing needle arthroscopy and conventional arthroscopy for false positives (FP).** The distribution appears scattered, reflecting low event rates and limited robustness.

Supplementary Figure 11: **Funnel plot comparing needle arthroscopy and conventional arthroscopy for false negatives (FN).** Interpretation is limited by small sample sizes and variability across studies.

Supplementary Figure 12: **Funnel plot comparing needle arthroscopy and conventional arthroscopy for sensitivity.** Some asymmetry is visible, likely reflecting substantial heterogeneity rather than true publication bias.

Supplementary Figure 13: **Funnel plot comparing needle arthroscopy and conventional arthroscopy for specificity.** The plot shows considerable dispersion, consistent with high between‐study heterogeneity.

Supplementary Figure 14: **Funnel plot comparing needle arthroscopy and conventional arthroscopy for KOOS4 at baseline.** The distribution appears symmetrical, suggesting no relevant small‐study effects.

Supplementary Figure 15: **Funnel plot comparing needle arthroscopy and conventional arthroscopy for KOOS4 at 0.5 months.** No evident asymmetry was observed, although the limited number of studies restricts interpretation.

Supplementary Figure 16: **Funnel plot comparing needle arthroscopy and conventional arthroscopy for KOOS4 at 1.5 months.** Interpretation is limited by the small number of studies and narrow range of effect sizes.

Supplementary Table 1: PRISMA Checklist.

## Data Availability

Available from corresponding author upon reasonable request.
