# Supplementary figures and images for: Needle arthroscopy as an emerging office‐based tool: Promising diagnostic value but limited evidence for therapeutic equivalence—A systematic review and frequentist meta‐analysis
Source: J Exp Orthop. 2026 Jul 30;13(3):e70857. doi: 10.1002/jeo2.70857 (PMC13420845; doi:10.1002/jeo2.70857)

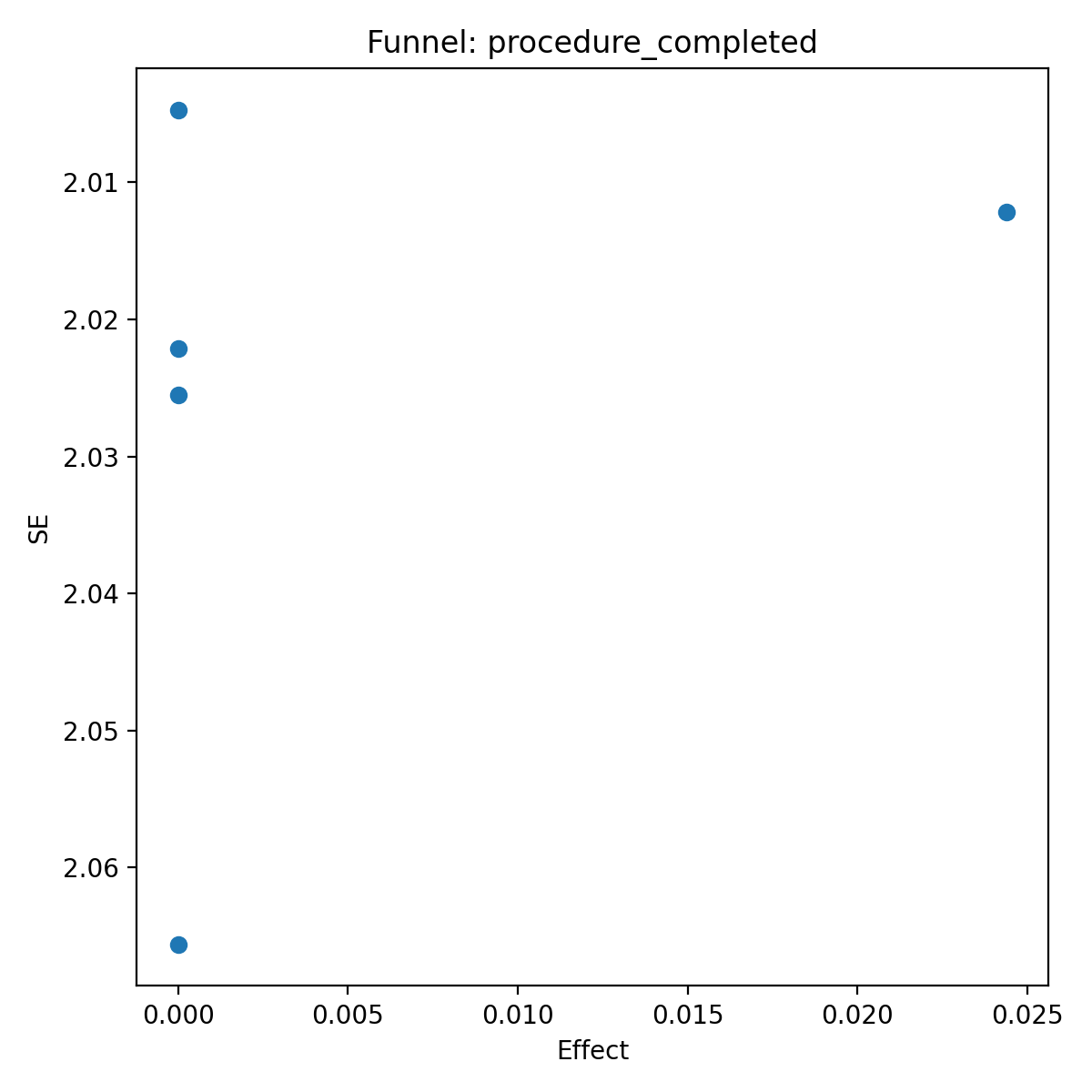

Supplement: Supplementary file 1 — Supplementary Figure 1: Funnel plot comparing needle arthroscopy and conventional arthroscopy for procedure completion rate. The distribution appears symmetrical, suggesting no relevant small‐study effects. [file JEO2-13-e70857-s004.png]

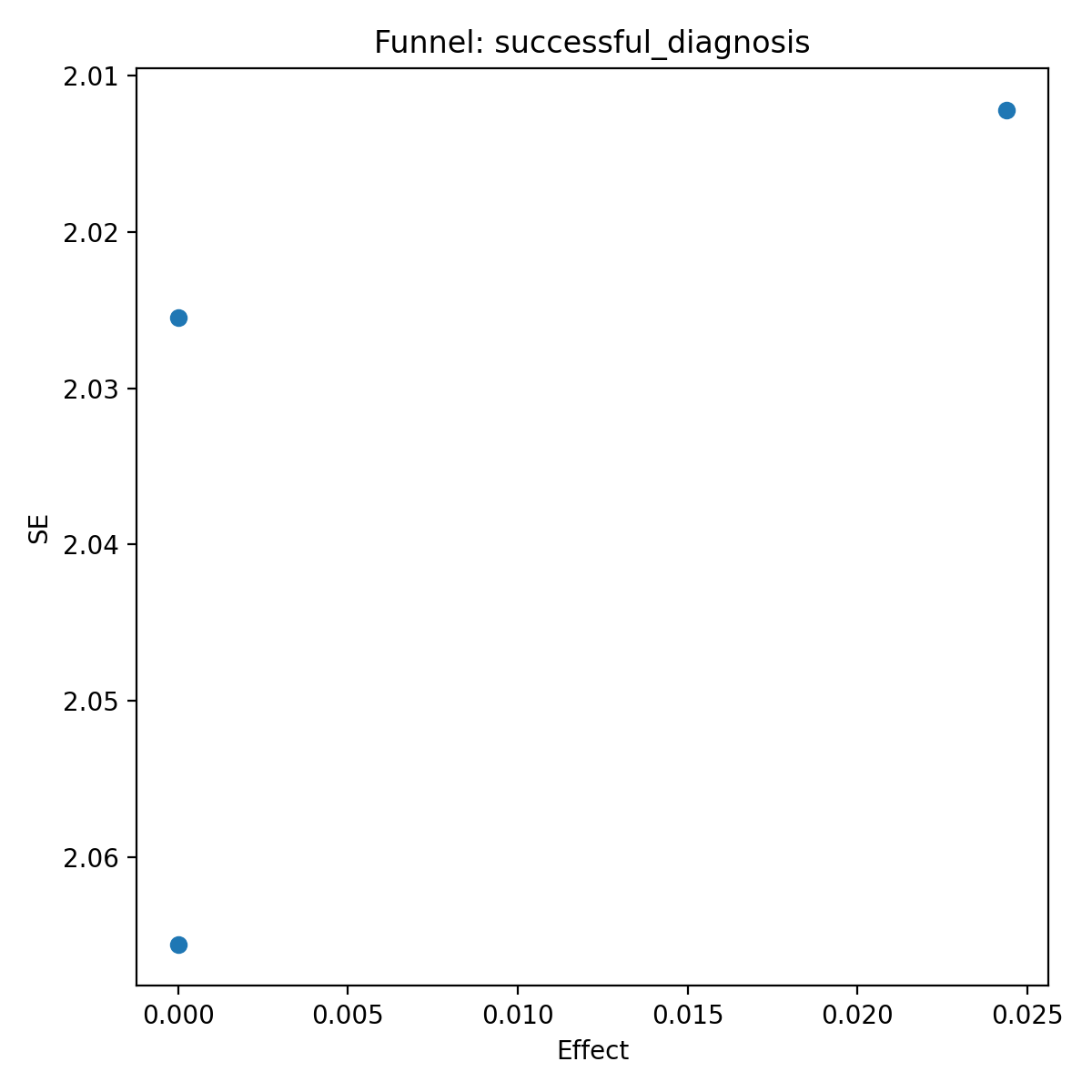

Supplement: Supplementary file 2 — Supplementary Figure 2: Funnel plot comparing needle arthroscopy and conventional arthroscopy for successful diagnosis. No clear asymmetry was observed, indicating a low likelihood of publication bias. [file JEO2-13-e70857-s010.png]

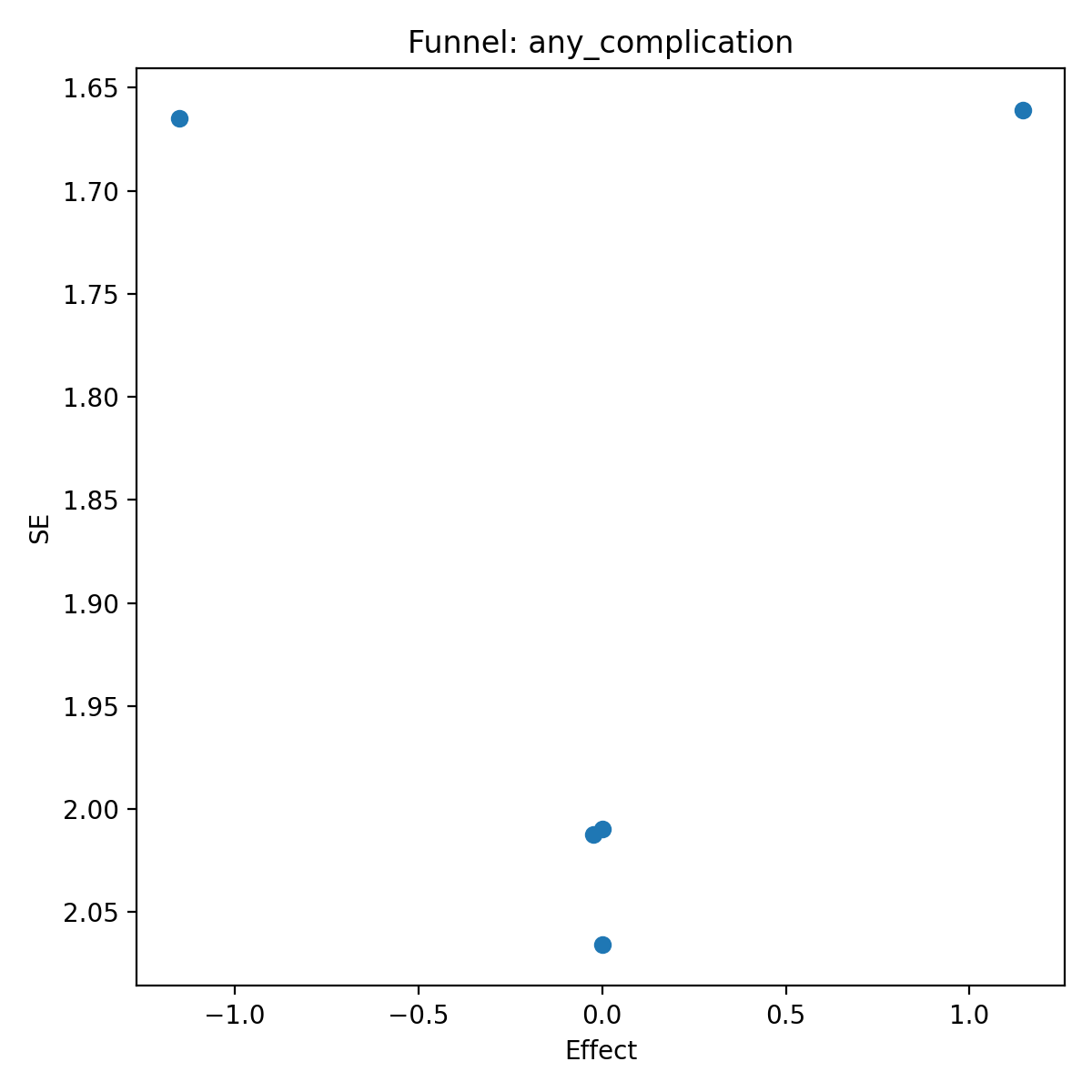

Supplement: Supplementary file 3 — Supplementary Figure 3: Funnel plot comparing needle arthroscopy and conventional arthroscopy for any complication. Interpretation is limited due to low event rates and a small number of studies. [file JEO2-13-e70857-s007.png]

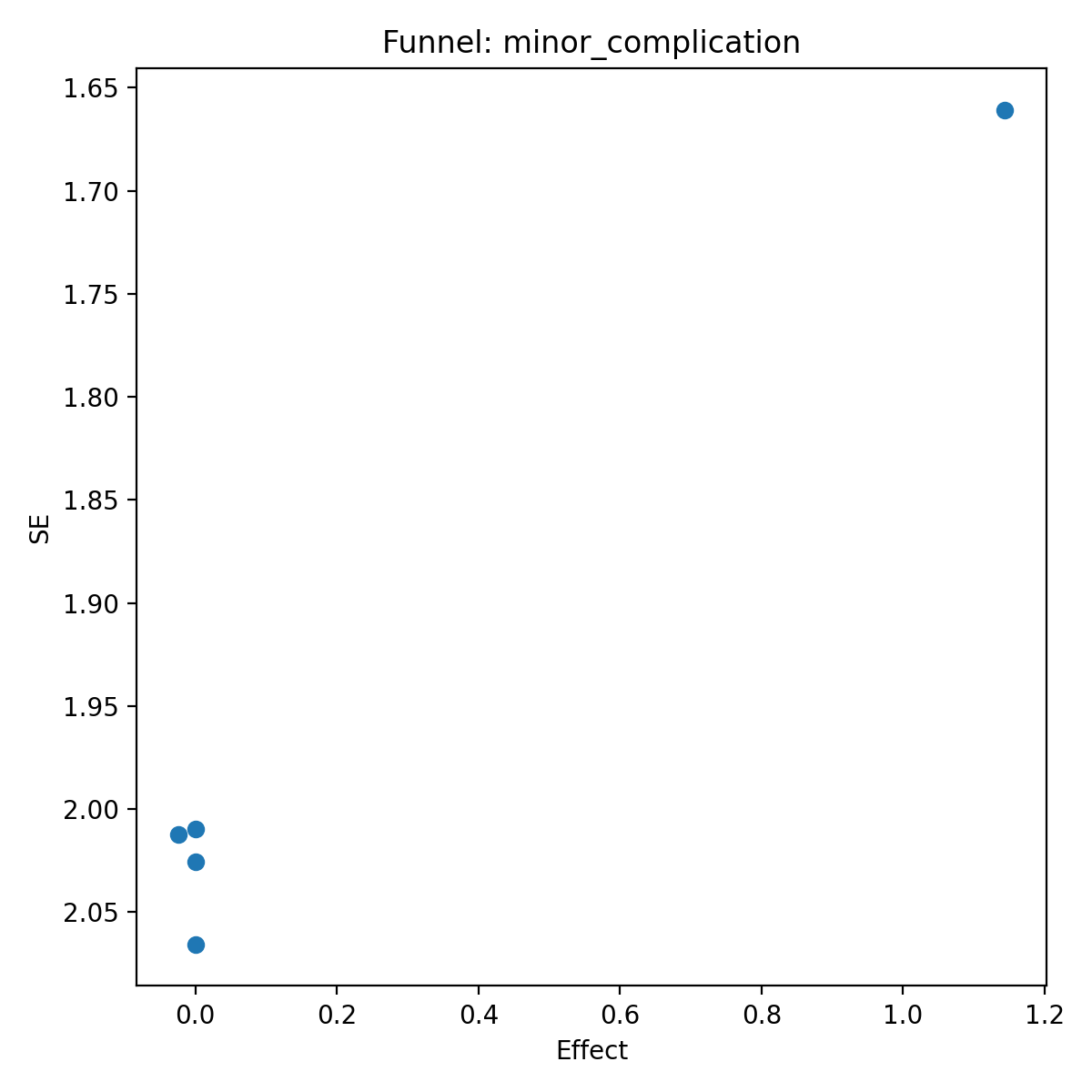

Supplement: Supplementary file 4 — Supplementary Figure 4: Funnel plot comparing needle arthroscopy and conventional arthroscopy for minor complications. The plot does not suggest substantial asymmetry, although conclusions are limited by sparse data. [file JEO2-13-e70857-s009.png]

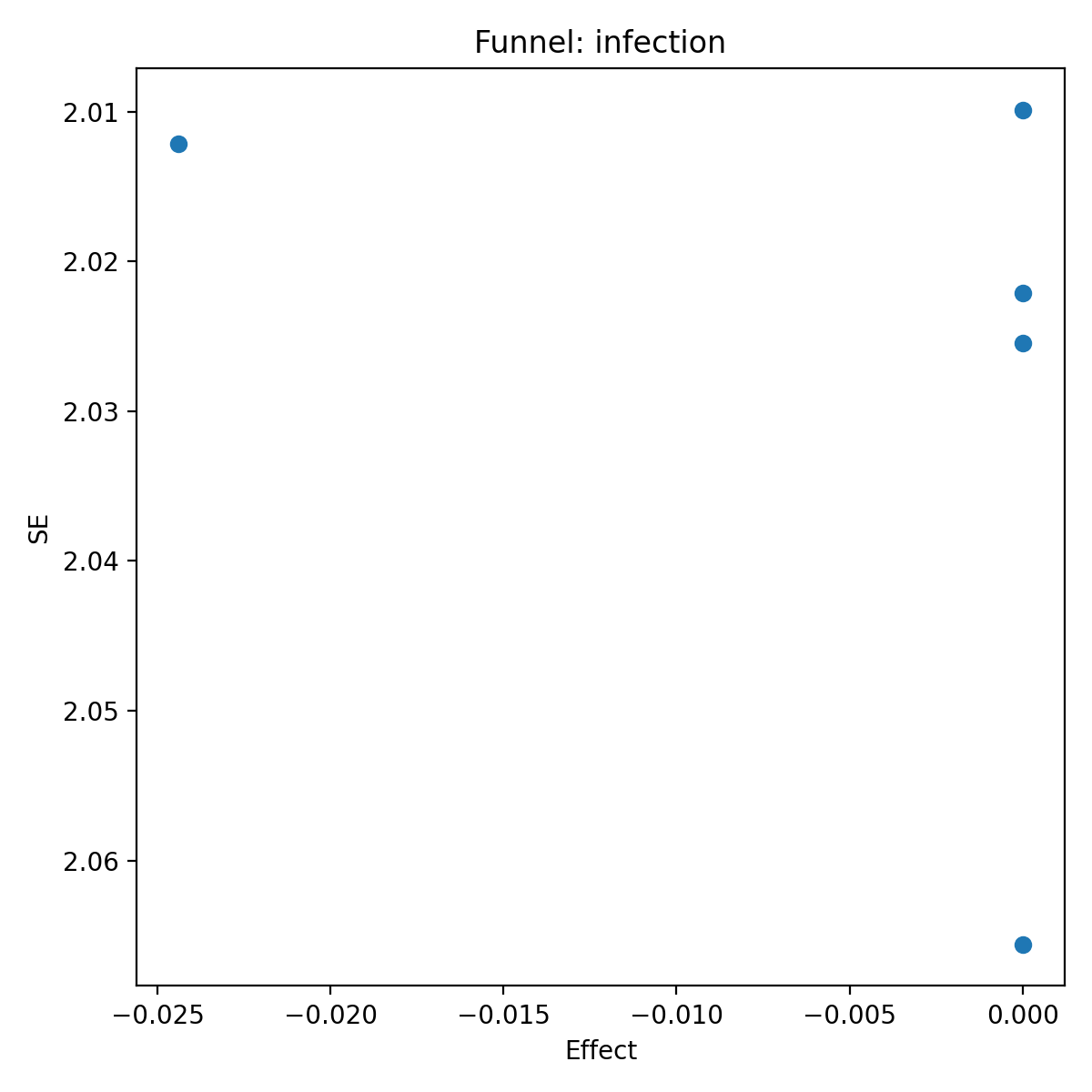

Supplement: Supplementary file 5 — Supplementary Figure 5: Funnel plot comparing needle arthroscopy and conventional arthroscopy for infection rates. Assessment is limited due to very low event counts and insufficient study numbers. [file JEO2-13-e70857-s017.png]

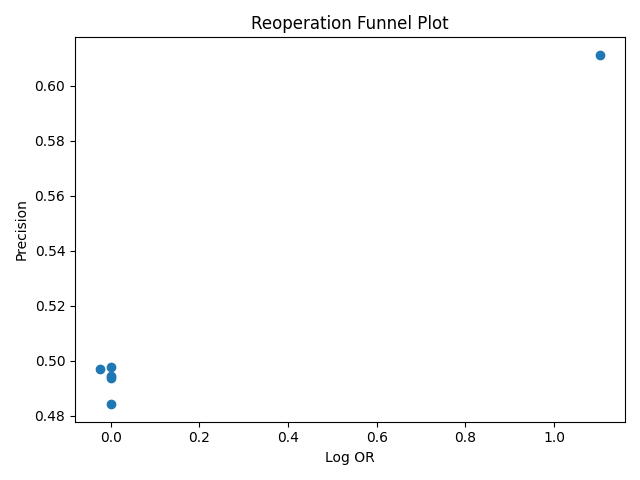

Supplement: Supplementary file 6 — Supplementary Figure 6: Funnel plot comparing needle arthroscopy and conventional arthroscopy for reoperation. Interpretation is not reliable due to the small number of studies and rare events. [file JEO2-13-e70857-s013.png]

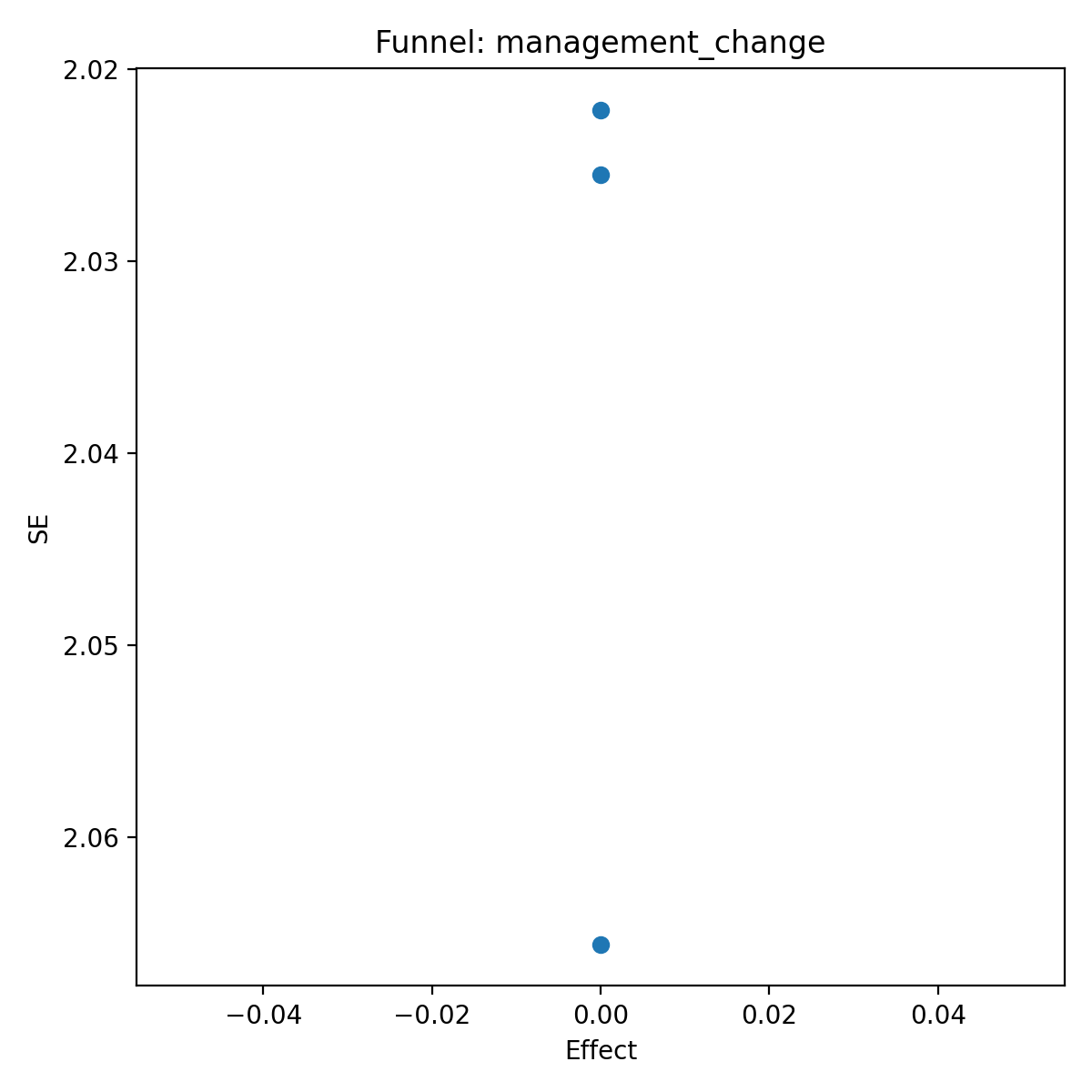

Supplement: Supplementary file 7 — Supplementary Figure 7: Funnel plot comparing needle arthroscopy and conventional arthroscopy for management change. No clear asymmetry was observed, but the small number of studies limits interpretation. [file JEO2-13-e70857-s006.png]

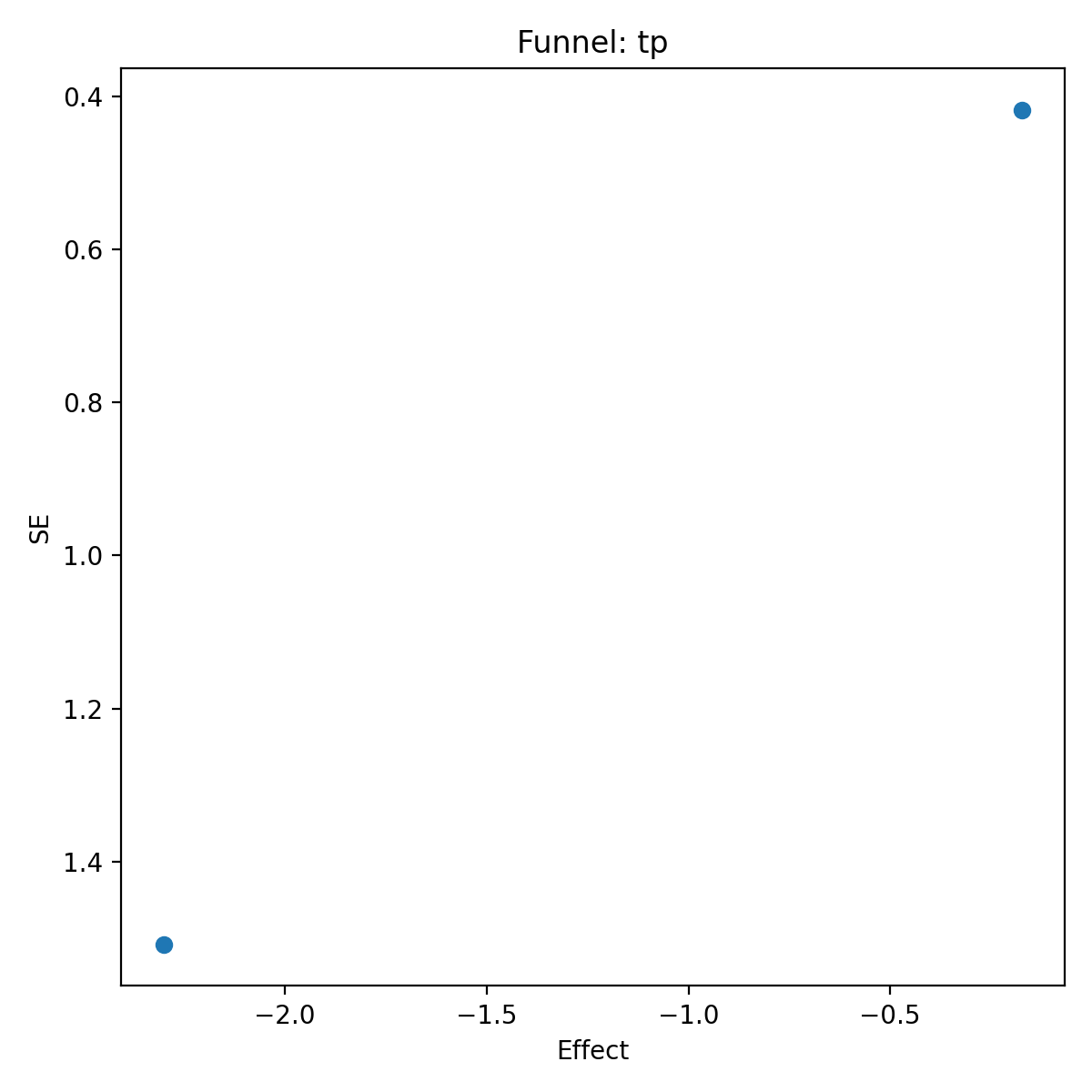

Supplement: Supplementary file 8 — Supplementary Figure 8: Funnel plot comparing needle arthroscopy and conventional arthroscopy for true positives (TP). Visual interpretation suggests variability across studies; however, results are based on reconstructed data and should be interpreted with caution. [file JEO2-13-e70857-s012.png]

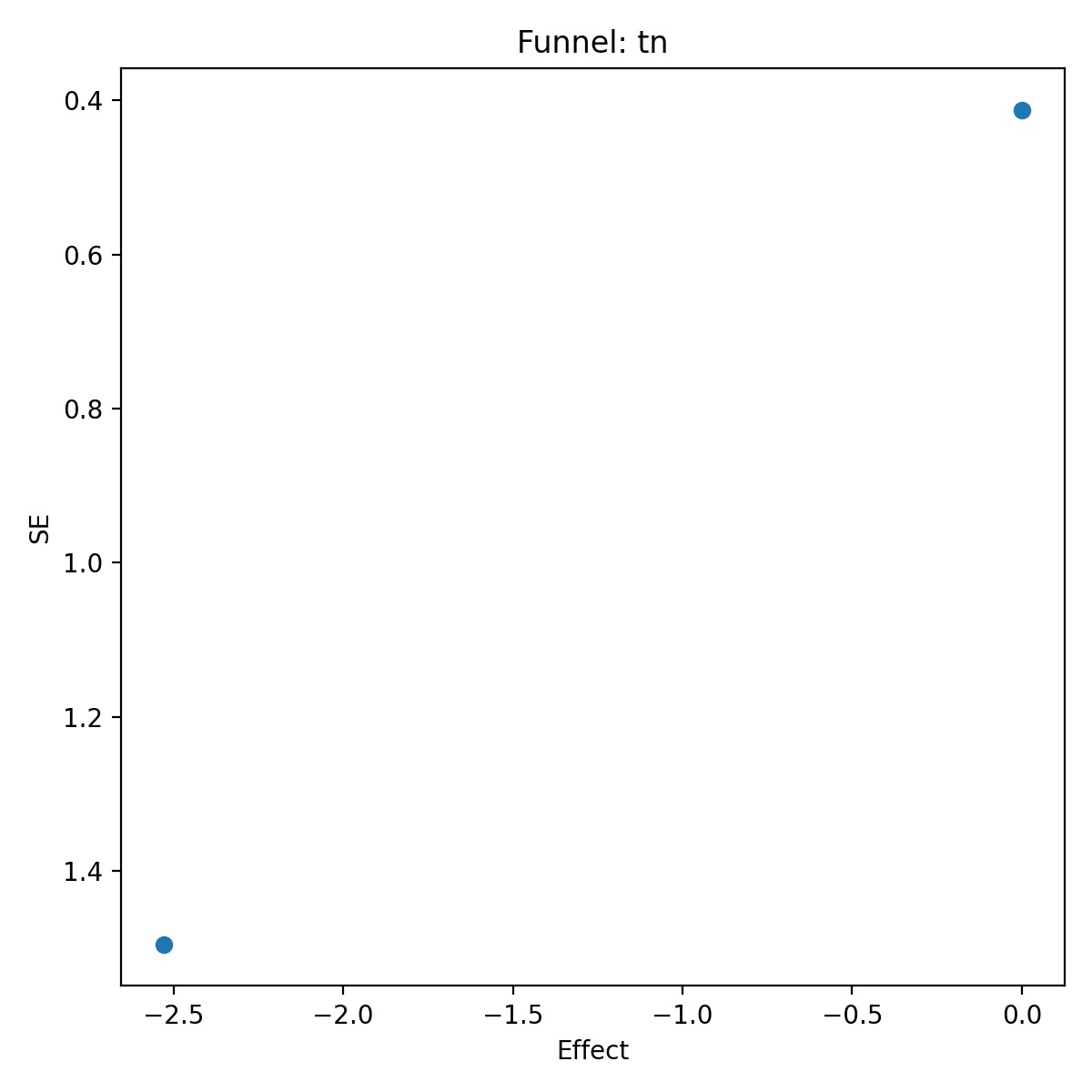

Supplement: Supplementary file 9 — Supplementary Figure 9: Funnel plot comparing needle arthroscopy and conventional arthroscopy for true negatives (TN). No clear pattern of asymmetry was observed, although precision is limited. [file JEO2-13-e70857-s001.png]

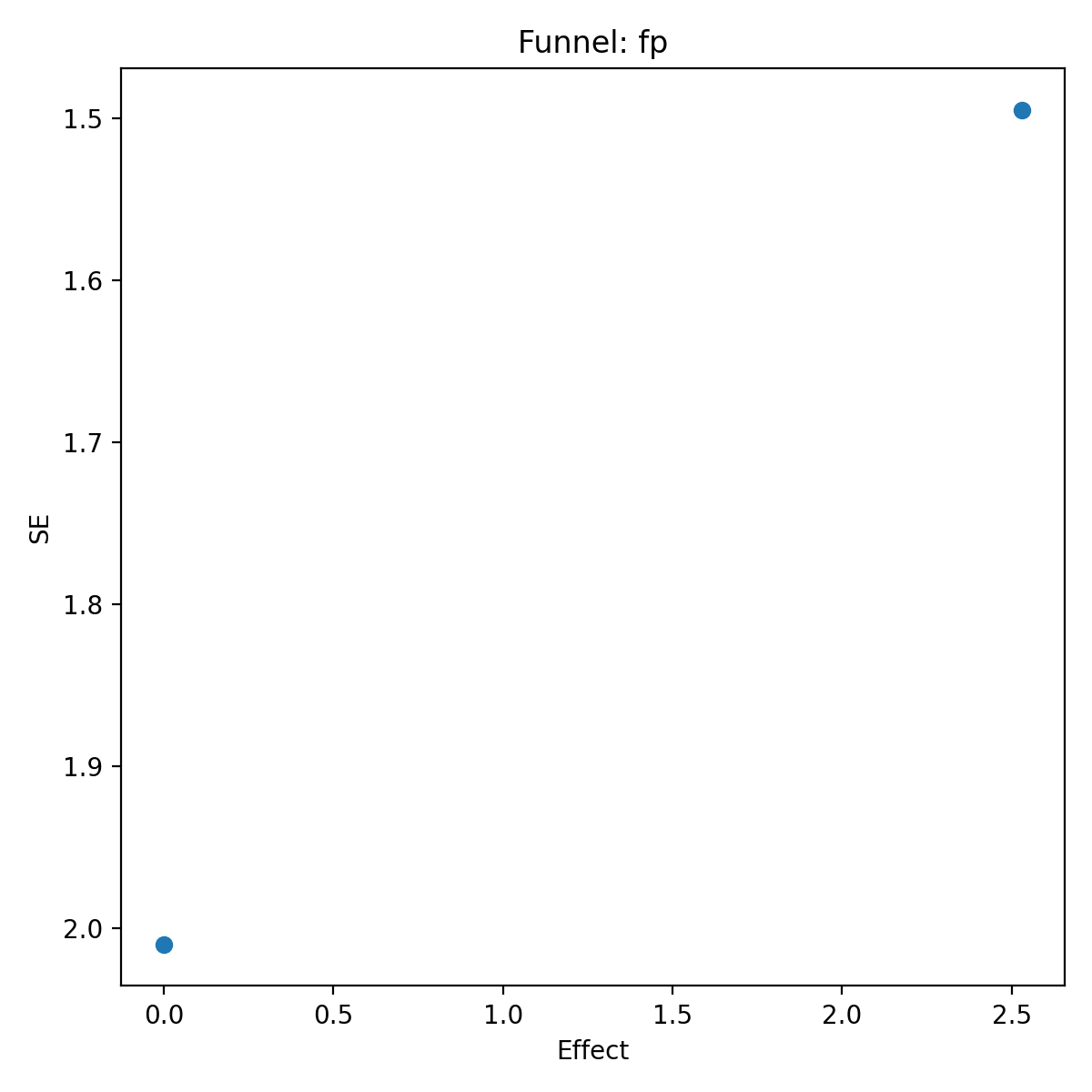

Supplement: Supplementary file 10 — Supplementary Figure 10: Funnel plot comparing needle arthroscopy and conventional arthroscopy for false positives (FP). The distribution appears scattered, reflecting low event rates and limited robustness. [file JEO2-13-e70857-s015.png]

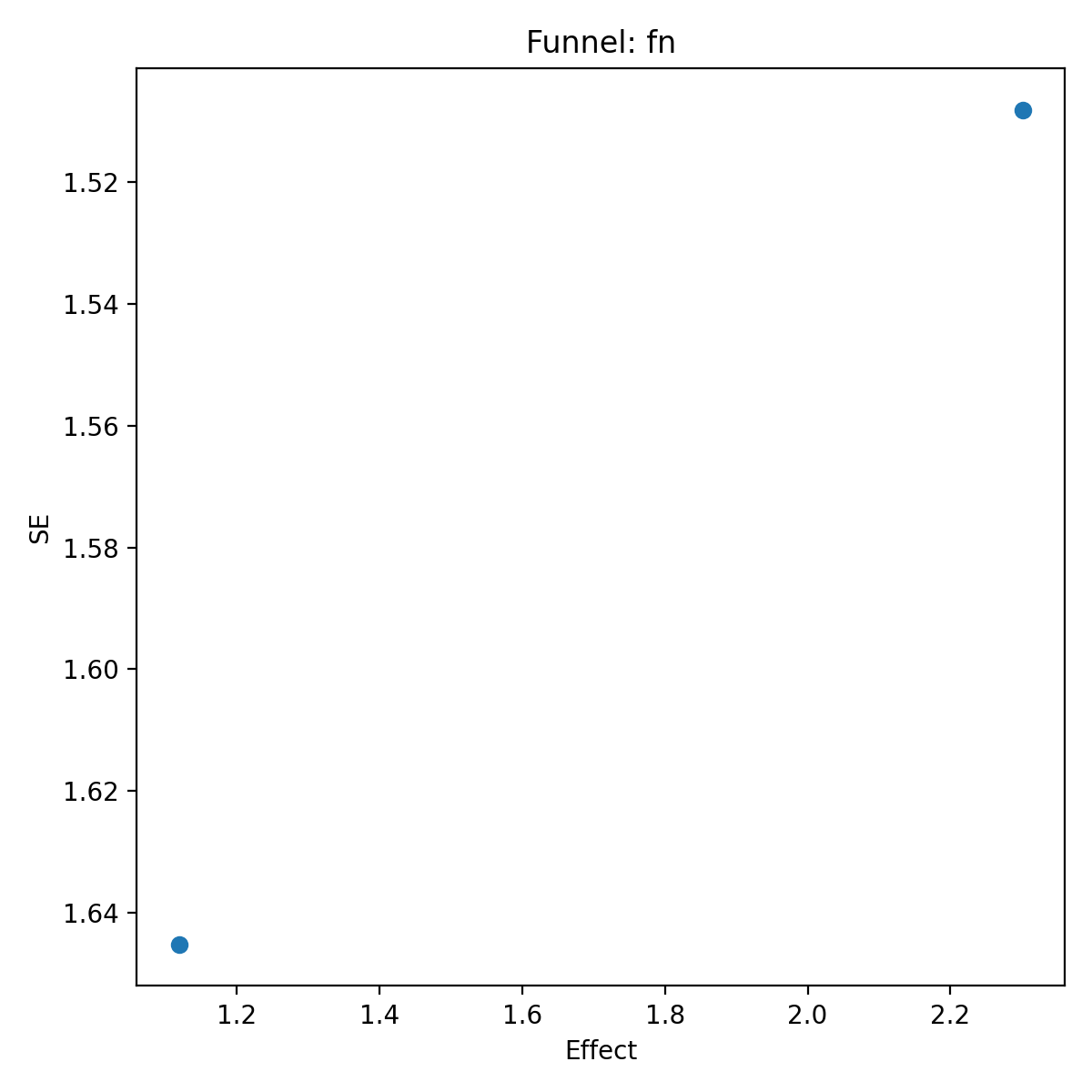

Supplement: Supplementary file 11 — Supplementary Figure 11: Funnel plot comparing needle arthroscopy and conventional arthroscopy for false negatives (FN). Interpretation is limited by small sample sizes and variability across studies. [file JEO2-13-e70857-s008.png]

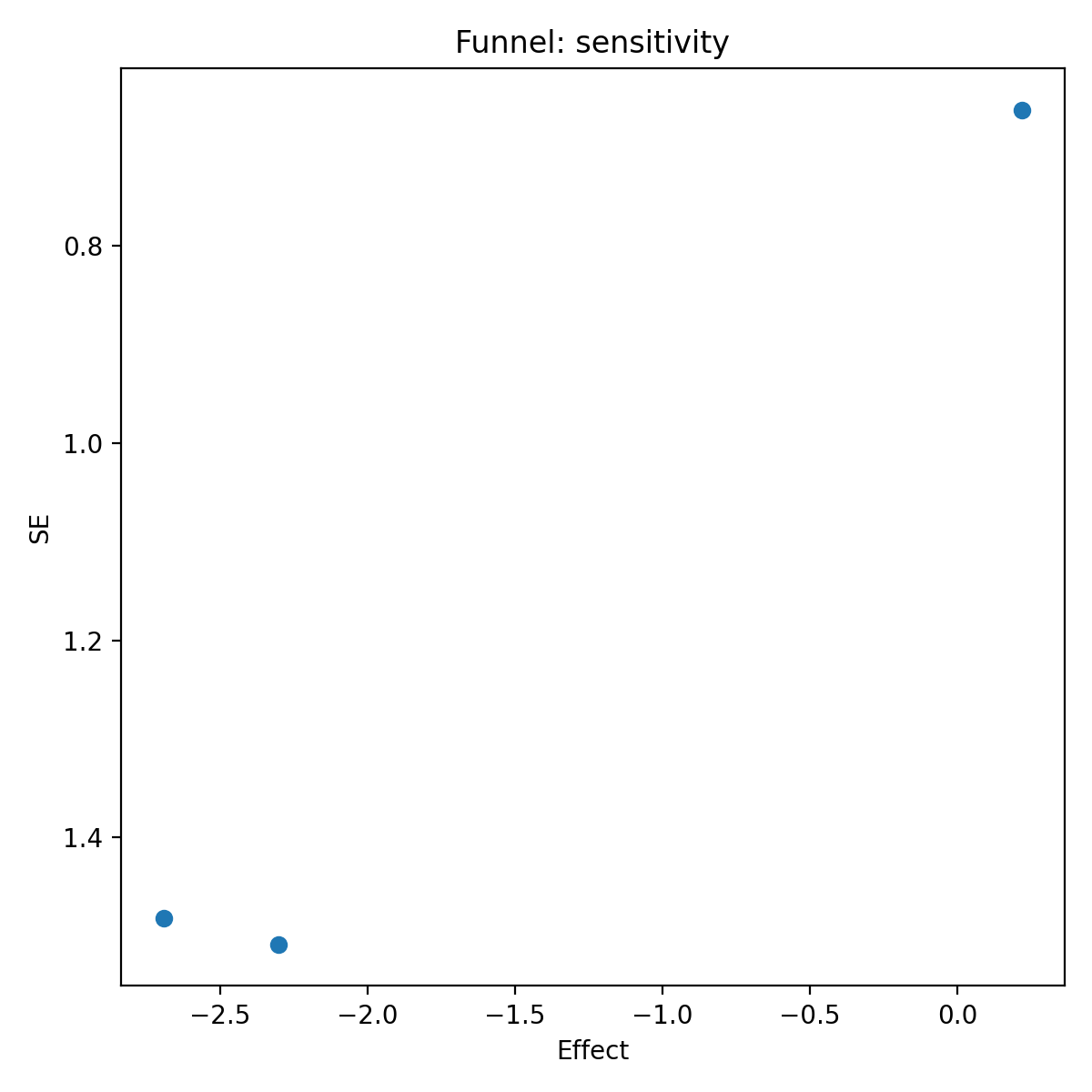

Supplement: Supplementary file 12 — Supplementary Figure 12: Funnel plot comparing needle arthroscopy and conventional arthroscopy for sensitivity. Some asymmetry is visible, likely reflecting substantial heterogeneity rather than true publication bias. [file JEO2-13-e70857-s002.png]

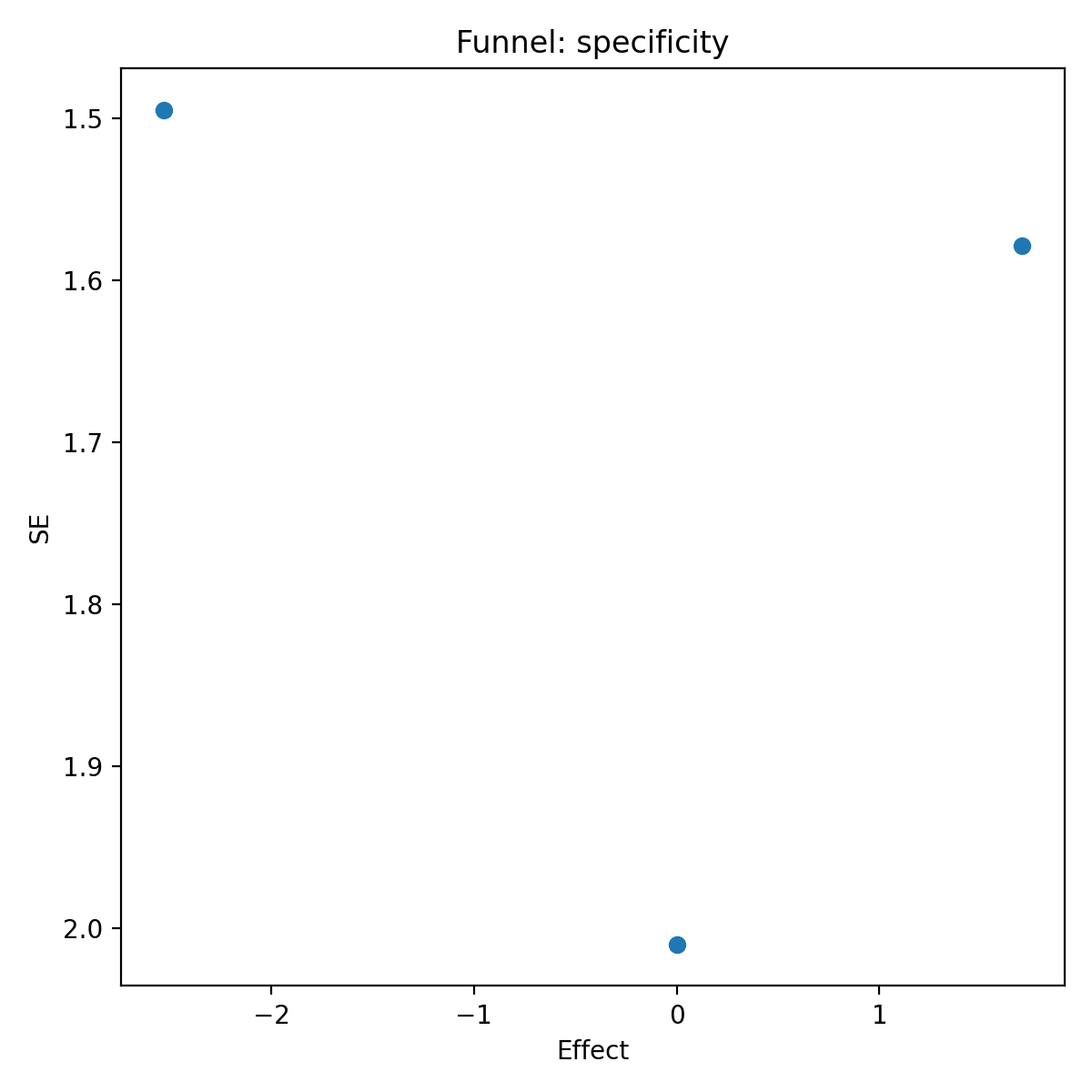

Supplement: Supplementary file 13 — Supplementary Figure 13: Funnel plot comparing needle arthroscopy and conventional arthroscopy for specificity. The plot shows considerable dispersion, consistent with high between‐study heterogeneity. [file JEO2-13-e70857-s003.png]

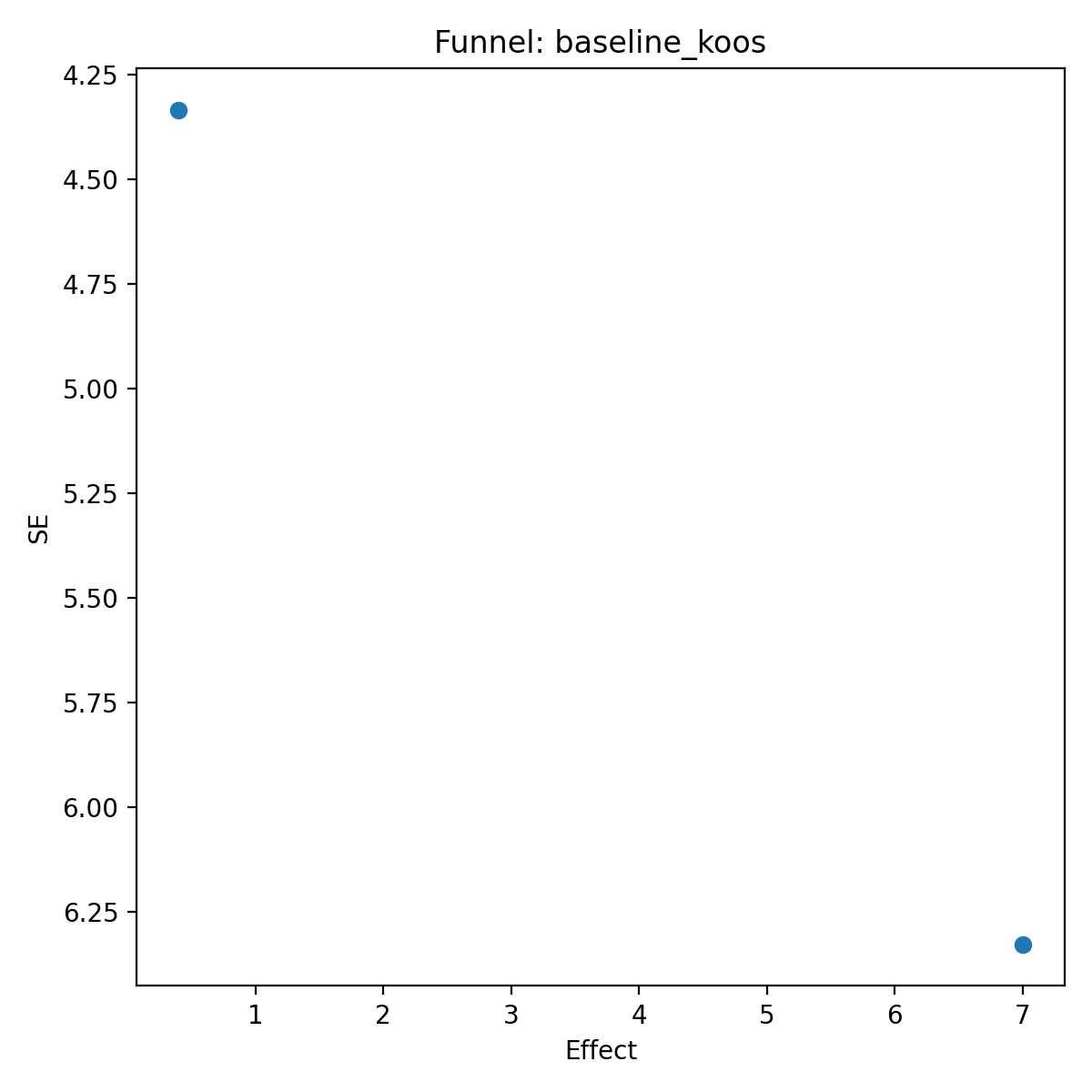

Supplement: Supplementary file 14 — Supplementary Figure 14: Funnel plot comparing needle arthroscopy and conventional arthroscopy for KOOS4 at baseline. The distribution appears symmetrical, suggesting no relevant small‐study effects. [file JEO2-13-e70857-s011.png]

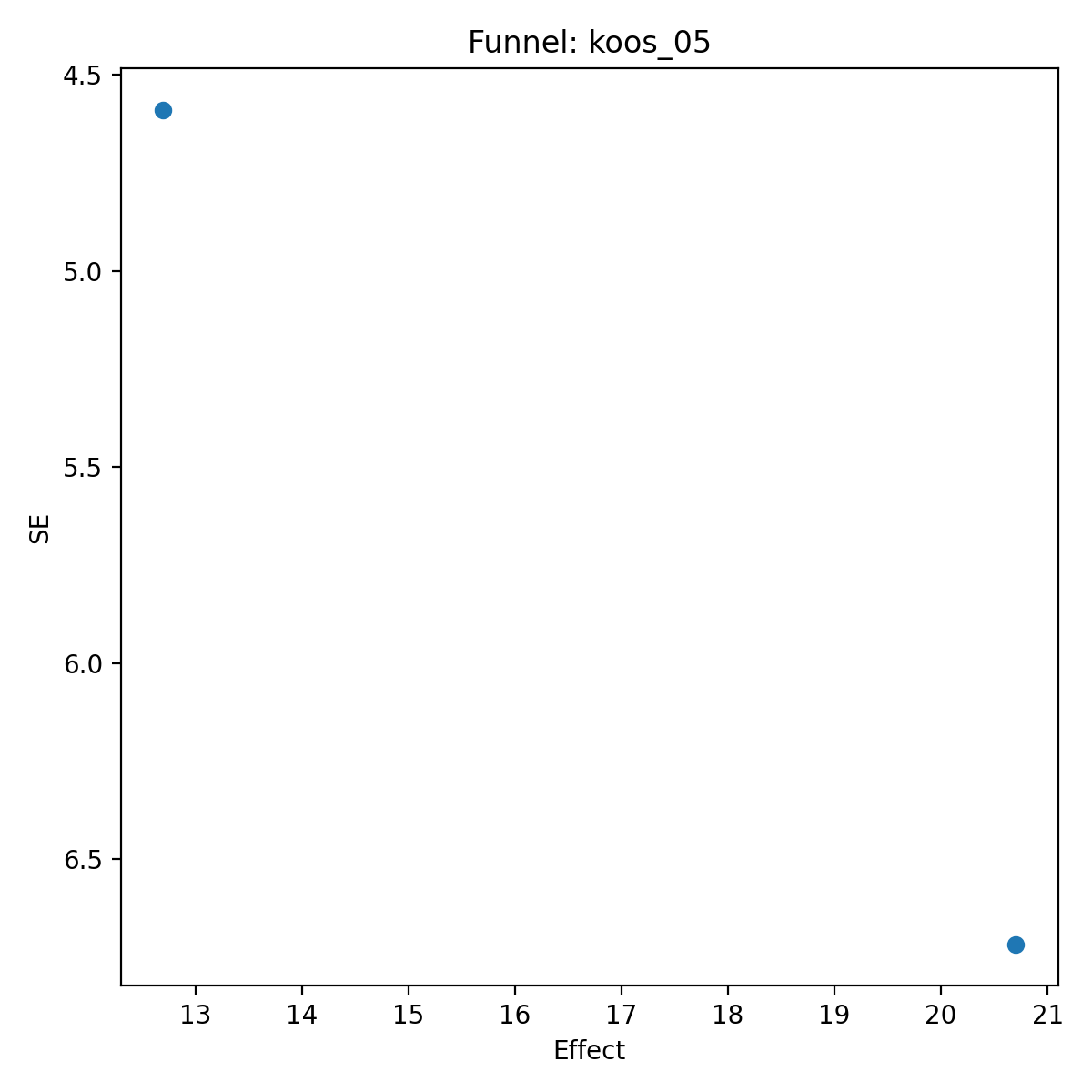

Supplement: Supplementary file 15 — Supplementary Figure 15: Funnel plot comparing needle arthroscopy and conventional arthroscopy for KOOS4 at 0.5 months. No evident asymmetry was observed, although the limited number of studies restricts interpretation. [file JEO2-13-e70857-s016.png]

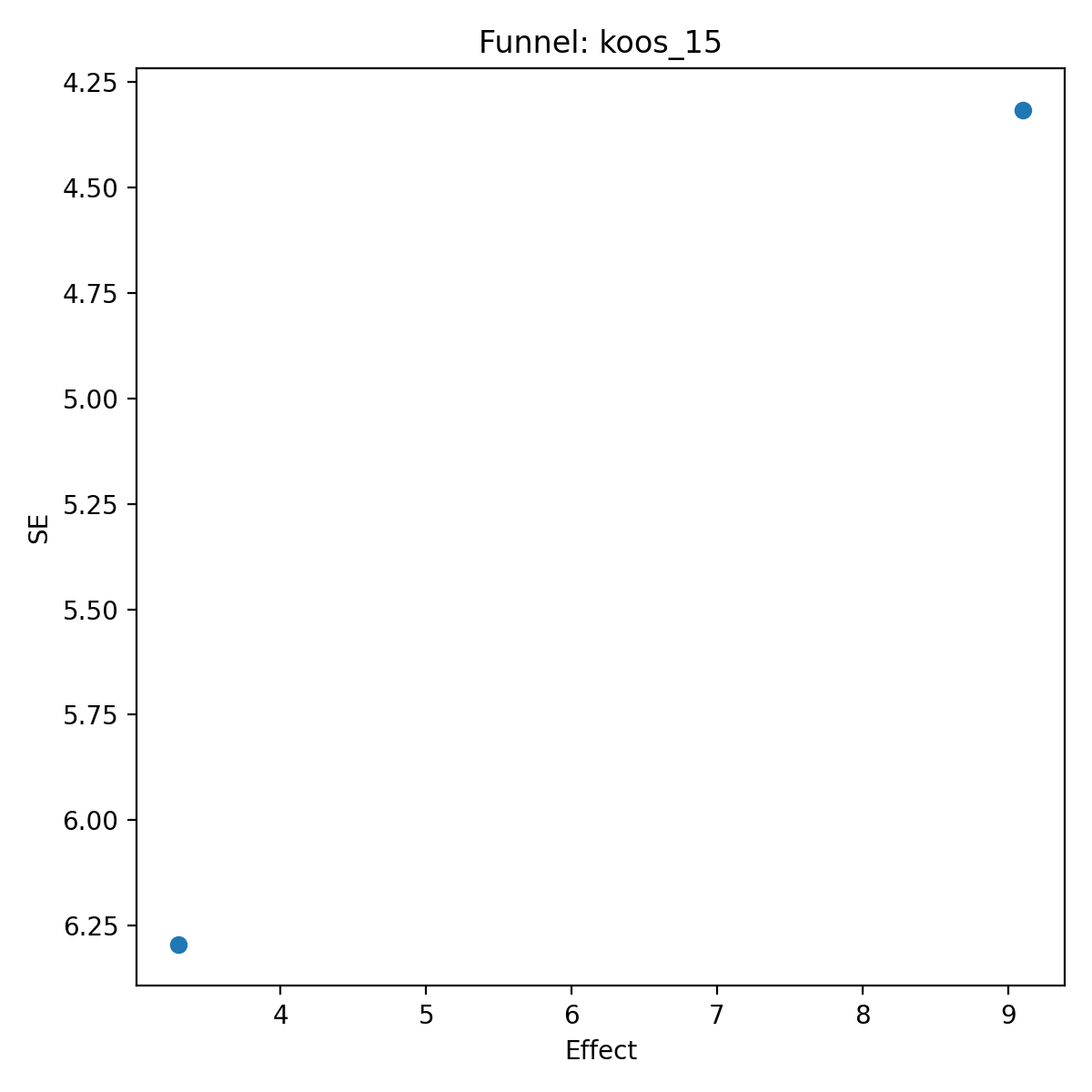

Supplement: Supplementary file 16 — Supplementary Figure 16: Funnel plot comparing needle arthroscopy and conventional arthroscopy for KOOS4 at 1.5 months. Interpretation is limited by the small number of studies and narrow range of effect sizes. [file JEO2-13-e70857-s005.png]
